# Gut Feelings: How Microbes, Diet, and Host Immunity Shape Disease

**DOI:** 10.3390/biomedicines13061357

**Published:** 2025-05-31

**Authors:** Benjamin Frederick Theis, Jun Sung Park, Jong Sung Anthony Kim, Sareh Zeydabadinejad, Matam Vijay-Kumar, Beng San Yeoh, Piu Saha

**Affiliations:** Department of Physiology and Pharmacology, University of Toledo College of Medicine and Life Sciences, Toledo, OH 43614, USA; benjamin.theis@rockets.utoledo.edu (B.F.T.); junsung.park@rockets.utoledo.edu (J.S.P.); jong.kim@rockets.utoledo.edu (J.S.A.K.); sareh.zeydabadinejad@utoledo.edu (S.Z.); matamvijay.kumar@utoledo.edu (M.V.-K.); bengsan.yeoh@utoledo.edu (B.S.Y.)

**Keywords:** fermentable fiber, short-chain fatty acids, probiotics, fecal microbiota transfer, circadian rhythm

## Abstract

The human gut microbiome is intricately linked to systemic and organ-specific immune responses and is highly responsive to dietary modulation. As metagenomic techniques enable in-depth study of an ever-growing number of gut microbial species, it has become increasingly feasible to decipher the specific functions of the gut microbiome and how they may be modulated by diet. Diet exerts both supportive and selective pressures on the gut microbiome by regulating a multitude of factors, including energy density, macronutrient and micronutrient content, and circadian rhythm. The microbiome, in turn, contributes to local and systemic immune responses by providing colonization resistance against pathogens, shaping immune cell activity and differentiation, and facilitating the production of bioactive metabolites. Emerging research has strengthened the connections between the gut microbiome and cardiometabolic disorders (e.g., cardiovascular disease, obesity, type-2 diabetes), autoimmune conditions (e.g., type-1 diabetes, rheumatoid arthritis, celiac disease), respiratory disease, chronic kidney and liver disease, inflammatory bowel disease, and neurological disorders (e.g., Alzheimer’s, Parkinson’s disease, depressive disorders). Here, we outline ways in which dietary factors impact host response in diseases through alterations of gut microbiome functionality and composition. Consideration of diet-mediated microbial effects within the context of the diseases discussed highlights the potential of microbiome-targeted treatment strategies as alternative or adjunct therapies to improve patient outcomes.

## 1. Introduction

In the 12th century, the physician and philosopher Maimonides wrote, “No disease that can be treated by diet should be treated with any other means” [1]. There has long been an understanding of the connection between diet and the maintenance of health, and while it is rarely the case that diet is relied upon as the primary treatment of modern diseases, recent research developments are shedding light on the nuanced role that diet can play in modulating disease outcomes. Beyond providing the macromolecules, nutrients, and energy necessary to sustain life, diet is an integral form of communication between the external environment and the host’s biological processes. This communication between dietary input and the host is not accomplished directly but instead interfaces via the activity of the trillions of microbes that inhabit the human gut [2,3]. The human microbiome, often referred to as a superorganism within our own bodies, facilitates digestion, metabolite production, maintenance of mucosal barriers, and maturation of the gut-associated lymphoid system [4,5]. The human gut microbiome primarily develops over the initial three years of life, with gradual exposure to diverse microbial species playing an essential role in educating the nascent immune system [6]. Diet impacts the microbiome from a very early age, with each major shift in infant microbiome composition being precipitated by dietary milestone events (e.g., transitioning from milk to solid foods) and the microbiome adapting to optimize nutrient utilization from various sources [7]. The mature human gut microbiome is primarily comprised of the phyla Firmicutes (Bacillota), Bacteroidetes (Bacteroidota), Actionobacteriota, Proteobacteria (Pseudomonata), and Verrucomicrobiota [8]. While Firmicutes and Bacteroidetes phyla typically account for more than 90% of human gut microbes, there is staggering diversity at finer taxonomic levels, with hundreds of families and thousands of unique species identified as common colonizers of the gut [9].

This wealth of data and the proliferation of metabolomic analysis techniques in the past 25 years has yielded a flurry of research unearthing associative relationships between microbiome composition and disease [2,10,11]. Shifts in gut microbiome functionality and composition, i.e., dysbiosis, have been shown to have significant effects on the disease etiology of autoimmune and inflammatory diseases, chronic obesity, diabetic conditions, neurological disorders, and infectious diseases. Microbiota exert their influence on disease progression through various mechanisms, including the production of secondary bile acids, microbial metabolites, and short-chain fatty acids (SCFAs), as well as by activating the transcription factors, membrane permeability, immune tolerance, production of inflammatory cytokines, and the circadian rhythms of host tissues [12,13,14,15,16]. These effects are not confined to the gut, as gut microbiota can also exert their effect systemically through a variety of pathways, such as the gut–liver and gut–brain axis [17,18].

In clinical applications, the modulation of microbiome composition can play a pivotal role in promoting immune response or serving as an adjuvant in support of other treatment modalities. Leveraging microbiome–disease relationships in treatment, however, is complicated by both the variability in microbiome composition between individuals and the diversity of metabolic pathways involved [19]. Established treatment methods, such as fecal microbiota transplantation (FMT) and live biotherapeutics, have potential applications in reversing dysbiotic conditions in various diseases; however, they have yet to be approved for clinical use outside of specific treatment cases such as recurrent gastrointestinal tract (GI) infection. Additionally, the efficacy of these therapies in peripheral use cases has been shown to be dependent on individual and dietary factors and encounters challenges in chronic diseases due to lack of recurrent treatment [20,21]. One step towards unpacking this complex relationship between the microbiome and disease is by gaining a better understanding of how dietary inputs modulate microbiome composition and functionality. Diet is the quintessential external factor responsible for shaping environmental conditions in the gut and is capable of inducing both short and long-term changes to the microbiome [22,23]. Although the gut microbiome’s response to dietary changes varies according to individual, cultural, and environmental factors, broad patterns have been identified. In this review, we provide an overview of the gut microbiome’s involvement in immune response, how dietary composition mediates microbial activity, and discuss how targeted dietary interventions may reshape the microbiome to enhance host immunity and ameliorate specific disease conditions.

## 2. The Gut Microbiota–Immune Axis: Key Players and Interactions

**Figure 1:**
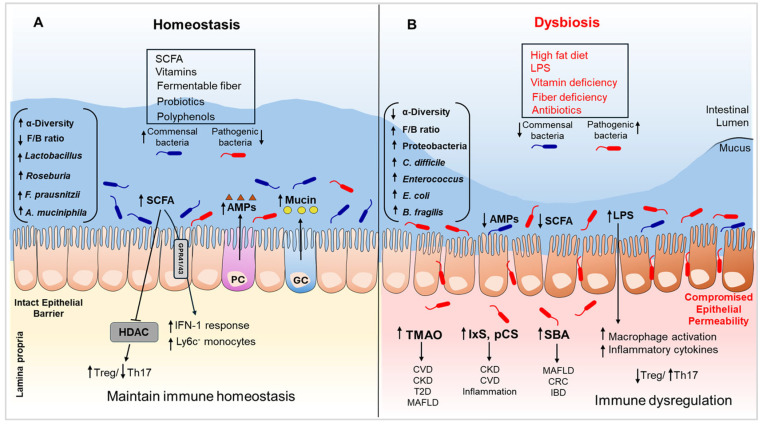
Gut homeostasis is maintained through the intricate interplay between the gut microbiota, dietary factors, and the immune system: (**A**) Gut homeostasis is maintained by a thriving population of beneficial (commensal) microbes and minimal presence of harmful (pathogenic) bacteria. The Firmicutes to Bacteroidetes (F/B) ratio is reduced in homeostasis conditions. These commensals contribute to gut health by generating key metabolites, including short-chain fatty acids (SCFAs), which support various physiological functions. In parallel, intestinal epithelial cells (particularly Paneth cells, PC) secrete antimicrobial peptides (AMPs) that help control pathogenic microbes and sustain microbial equilibrium. Goblet cells (GC) produce mucin, a critical component for maintaining intestinal barrier integrity and permeability. The gut lining consists of epithelial cells, forming a barrier that restricts the movement of luminal substances and microbes. Beneath this epithelial layer lies the lamina propria, densely populated with immune cells. SCFAs contribute to immune regulation by inhibiting the histone deacetylase (HDAC) pathway, thereby helping to balance both innate and adaptive immune responses. Dietary components such as vitamins, fermentable fibers, probiotics, and polyphenols also play a key role in maintaining gut homeostasis and supporting immune surveillance. (**B**) Gut microbiota dysbiosis occurs when beneficial microbes reduce and pathogenic species proliferate. The F/B ratio is increased in dysbiosis conditions. This disruption can result from various factors, including dietary imbalances, such as high-fat diets, deficiencies in vitamins or dietary fiber, and increased intake of microbial toxins like lipopolysaccharides (LPS), as well as antibiotic use and other external or genetic influences. As dysbiosis advances, the production of SCFAs and AMPs is reduced, compromising the integrity of the intestinal epithelial barrier. This weakened barrier allows pathogenic microbes and microbial products to translocate into the lamina propria. In response, resident immune cells trigger an inflammatory cascade (increased macrophage activation, elevated Th17 cells, and lowed Tregs), recruiting additional immune cells and promoting tissue inflammation. Within this figure (↑) and (↓) indicates increase and decrease respectively, in the abundance of the microorganisms, metabolites, or cytokines listed. Certain microbial metabolites, including trimethylamine N-oxide (TMAO), indoxyl sulfate (IxS), p-cresyl sulfate (pCS), and secondary bile acids (SBA), are implicated in the pathogenesis of various chronic diseases such as cardiovascular disease (CVD), chronic kidney disease (CKD), type 2 diabetes (T2D), metabolic-associated fatty liver disease (MAFLD), inflammatory bowel disease (IBD), and colorectal cancer (CRC).

The gut microbiota comprises trillions of microorganisms, including bacteria, viruses, and fungi, that occupy a specific niche in synergy with the host [24]. The microbiota interacts with host immune cells to maintain immune homeostasis and influence immune responses through various mechanisms: (i) direct interactions with intestinal epithelia; (ii) modulation of immune cell functions; (iii) the production of bioactive metabolites like SCFAs, secondary bile acids, and indole. These processes are crucial for maintaining mucosal immunity, regulating systemic immune responses, and preventing immune-related disorders. The interaction between the gut microbiota and the intestinal epithelia produces continuous immune signaling [25]. The regulation of this immune response, alongside the maintenance of epithelial barrier integrity and permeability, is crucial for intestinal homeostasis [26]. The intestinal epithelia, as accessory immune cells, are vital mediators of intestinal homeostasis that maintain an immunological environment permissive to colonization by commensal bacteria and protect against invasion from pathogenic microorganisms [27] (Figure 1). The epithelial barrier is comprised of several components: (i) The outer mucus layer surrounds the commensal microbiota and contains antimicrobial proteins (AMPs) and secretory immunoglobulin A (sIgA). (ii) The central epithelial layer consists of various types of cells like epithelial cells, goblet cells (secrete mucins), Paneth cells (synthesize AMPs), enterocytes (absorb water and nutrients), enteroendocrine cells (produce hormones), and microfold cells (i.e., M cells, specialized for antigen presentation). (iii) The inner layer consists of lamina propria (LP), which includes both innate (e.g., macrophages, natural killer cells) and adaptive (T cells, B cells), providing an immune-ready environment to respond to potential threats [28]. In the gut, mucosal immune cells are either present in gut-associated lymphoid tissues (GALT), where they carry the antigen-specific adaptive immune responses, or accumulate in the LP as a network of innate and adaptive effector cells. Organized GALT consists of various lymphoid follicles, like mesenteric lymph nodes, Peyer’s patches (PPs), and isolated lymphoid follicles (ILFs) [28].

Gut microbiota plays a critical role in shaping the activity and responsiveness of immune cells, influencing their ability to mount effective immune responses to pathogens or modulate inflammation. The interactions between the host immune system and microbiota are mediated through the recognition of pattern-recognition receptors (PRRs). PRRs are a diverse family of extracellular and intracellular receptors that detect specific microbe-associated molecular patterns (MAMPs). These receptors include Toll-like receptors (TLRs), nucleotide-binding oligomerization domain (NOD)-like receptors (NLRs), and C-type lectin receptors (CLRs) [29]. Upon activation, PRRs trigger the induction of chemokines and cytokines that orchestrate a protective immune response. TLRs play a vital role in regulating several key processes in the gut immune response. They actively contribute to the secretion of mucus and AMPs, which are essential for maintaining the intestinal barrier and defending against pathogens. TLR activation also promotes IgA class switching, which enhances mucosal immunity by producing secretory IgA antibodies that opsonize bacteria in the lumen, thus preventing their access to the LP. Furthermore, TLRs induce the expression of the polymeric immunoglobulin receptor, facilitate the translocation of tight junction proteins to strengthen the intestinal epithelial barrier, and stimulate the expression of nicotinamide adenine dinucleotide phosphate (NADPH) oxidase. This leads to the production of reactive oxygen species (ROS), which further support the immune defense against pathogens [30,31,32]. Studies have shown that *Bacteroides fragilis* (*B. fragilis*) activates the TLR pathway of T lymphocytes to establish host-microbial symbiosis and influences T cell development and differentiation [33,34]. MyD88 is the key signaling adapter protein in the innate immune response, acting as a central intermediary between TLRs and downstream signaling pathways. Most TLRs utilize MyD88 for signaling, except for TLR3 [35]. In addition, MyD88-dependent signaling pathways are imperative for the activation of NF-κB, leading to the production of inflammatory cytokines and the activation of immune responses. MyD88 deficiency results in an impaired innate immune response and increased susceptibility to infections [36,37]. Study shows that MyD88 signaling in T cells controls IgA-mediated control of the microbiota [38].

Adaptive immune cells (T and B lymphocytes) play a pivotal role in the maintenance of immune homeostasis by modulating antigen-specific responses and supporting the structural and functional integrity of the barrier functions of the gut mucosa. Gut microbiota can modulate the differentiation of T cells into distinct subsets, such as T-helper (Th) cells (Th1, Th2, Th17) or regulatory T cells (Tregs) [39]. Conversely, gut dysbiosis can disrupt this immune balance, leading to aberrant T cell responses that can contribute to the development of immune-mediated disorders [40]. Moreover, intricate alterations occur in T cells with increasing age, including epigenetic and metabolic disorders, which can affect naive, memory, and effector T cell populations [41,42]. Furthermore, the T cell receptor (TCR) repertoire diminishes with age, and this is accompanied by an increase in the frequency of senescent or exhausted T cells, which are functionally inactive [43]. This immunosenescence contributes to the impaired immune system and heightens the risk of chronic inflammation. Butyrate, a short-chain fatty acid (SCFA) produced by commensals, plays a significant role in counteracting this process by promoting the differentiation of peripherally induced Tregs, which in turn inhibits the development of systemic inflammation [44]. In addition to butyrate, de novo Treg-cell generation was also potentiated by propionate but not by acetate [44]. SCFAs, in general, are also capable of reprogramming the metabolic activity of immune cells, leading to the induction of regulatory B cells (Breg) and enhancing IL-10 (a pleiotropic cytokine) production and suppressing Th17 cells, which are important processes in controlling the inflammatory bowel diseases (IBD) and autoimmune disorders [45]. In the context of host–pathogen interactions, commensal activation of memory T cells and trafficking to inflamed sites is critical for protecting against bacterial infections. IL-10-mediated anti-inflammatory responses by commensals also play a protective role in inflammatory conditions. Additionally, studies have shown that microbiota-derived ATP promotes the differentiation of lamina propria Th17 cells [46], bacteria-derived tryptophan metabolites can induce intraepithelial CD4^+^ CD8^+^ T cells, and bacterial polysaccharides can expand Tregs [47]. In vivo studies have demonstrated that *Bifidobacteria* species play crucial roles in regulating immune responses, including balancing Th1 and Th2 responses, promoting Th17 cell polarization, and activating CD8^+^ T cell effectors. In mouse models, specific *Bifidobacteria* species have been shown to potently induce the development of intestinal Th17 cells [48,49]. For instance, mice fed with *Bifidobacterium longum* exhibited a significant increase in CD4^+^ T cells and a decrease in CD4^+^CD8^+^ T cell levels in mesenteric lymph nodes and Peyer’s patches [50]. Additionally, *Bifidobacteria* has been implicated in tumor inhibition. Studies have demonstrated that *Bifidobacterium*-treated mice displayed significantly improved tumor suppression compared to untreated counterparts [51], suggesting a potential role for these bacteria in modulating anti-tumor immune responses. Moreover, *Bifidobacterium*-treated mice exhibited significantly improved tumor suppression, with efficacy comparable to anti–PD-L1 therapy. Notably, the combination of *Bifidobacterium* treatment and checkpoint blockade produced a synergistic effect, resulting in enhanced control of tumor growth [51].

Interactions between the gut microbiota and gut-associated lymphoid tissue (GALT) are essential to the regulation of B cell maturation, activation, and functions within the intestinal immune environment. B cells are important for adaptive immunity due to their capacity to differentiate into antibody-producing plasma cells that generate a substantial amount of IgA antibodies, which are released within the mucosa. Gut commensals determine the type of IgA response: pathobionts with invasive properties induce T cell-dependent IgA responses, whereas most bacteria induce T cell-independent IgA responses [52]. IgA plays a crucial role in maintaining gut homeostasis by neutralizing pathogens, preventing bacterial adhesion to the intestinal epithelium, and modulating immune responses to commensal microbes; therefore, any disbalance in IgA response will impact the microbial abundance and diversity in the gut mucosa, leading to dysbiosis [53]. Within the GALT, B cells undergo class switching to IgA in response to microbial and dietary antigens, a process influenced by cytokines such as TGF-β [54]. TGF-β contributes to maintaining gut microbiota and immune tolerance-promoting IgA production by B cells, which in turn creates a barrier that restricts the adhesion, growth, and entry of potential pathogens and their harmful derivatives [40]. Moreover, B cells play an important role in immune regulation by interacting with T cells, antigen-presenting cells (APCs, like DCs), and releasing secretary cytokines. Bregs suppress excessive inflammation through IL-10 production and maintain immune homeostasis [55], thus preventing immune-mediated diseases such as IBD and other autoimmune conditions.

## 3. Mechanisms Linking Diet, Gut Microbiota, and Immunity

### 3.1. Macronutrients

Every aspect of gastrointestinal function, from nutritional absorption to metabolic activity to immunity, is influenced by the gut microbiome, and the gut microbiome is similarly subject to dietary modulation. The gut microbiome is not just a beneficiary positioned to harvest nutrients from our diet but an indispensable contributor that enables efficient adaptation to dietary changes and facilitates energy harvest and storage. Beginning broadly, the macronutrient composition of the diet dictates the most basic environmental conditions for the gut microbiome. Simple ecological mechanisms have been proposed to underpin the response of the gut microbiome to dietary intake, whereby energy density and nutrient scarcity apply the primary selective pressure on the gut microbiome [56]. Interestingly, dietary protein and carbohydrate intake have been found to have a significantly greater impact on gut microbial abundance and diversity than dietary fat intake. Reduced dietary protein intake intensifies microbial competition for nitrogen, leading to a compositional shift toward a bacterial community characteristic of nitrogen-limited conditions, referred to as a ‘limitation-type’ bacterial cohort [56]. Microbial taxa associated with this low-energy-density, low-protein diet demonstrate associations with improved intestinal function and are also capable of metabolizing proteoglycans from the host’s mucosal surface [57]. Researchers have proposed a feedback-loop model in which reductions in dietary nitrogen availability resulted in improved intestinal functions, increasing the availability of endogenous nitrogen sources to support microbes that can thrive in limited nitrogen conditions. This model enabled the classification of broad microbial cohorts based on their response to nutrient scarcity without accounting for ecological interactions across higher taxonomic levels. While macronutrient availability imposes fundamental selective pressure that shapes the gut microbiota, the complexity of these interactions is further amplified by the distinct substrate utilization of bacterial taxa across different phyla.

Allocation of daily calories between macronutrient groups dictates the environmental conditions for the gut microbiome. High protein diets, which are often low in carbohydrate and fiber content, have been shown to decrease the levels of beneficial fiber-derived metabolites such as butyrate and shift the gut microbiome composition toward dysbiosis [58,59,60]. Conversely, when paired with calorie restriction, high protein diets have been found instead to have positive effects on microbial diversity [61]. Furthermore, the source of dietary protein, whether animal or plant-based, has been shown to distinctly influence the composition and diversity of the gut microbiome [22,62]. From an energetics perspective, gut microbiota tends to favor carbohydrate metabolism as a primary source of energy, often reserving amino acids for anabolic processes to avoid energy-intensive amino acid biosynthesis [63]. This is further evidenced by the gross abundance of unique carbohydrate-activating enzymes in microbial genomes [64,65]. Humans are highly reliant on the carbohydrate-degrading function of gut microbes, as the human genome encodes relatively few enzymes for complex carbohydrate breakdown. Indeed, the structural form and complexity of dietary carbohydrates significantly affect the outcomes of microbial fermentation and the composition of gut microbiota.

Resistant starches (RS) and dietary fiber, both composed of complex carbohydrates and resistant to human digestion, are critical in maintaining the function of the gut microbiome. RS passes through the small intestine in an undigested form to selectively stimulate saccharolytic microbes and reduce the production of potentially harmful branched-chain amino acids (BCAAs) [66,67]. Similarly, fiber-rich diets enhance microbiome functionality by upregulating microbiome activity, providing precursors for SCFA synthesis, and downregulating BCAA production [68,69,70,71]. Conversely, chronic dietary fiber deficiency results in a significant reduction in microbial diversity with effects that can accumulate across generations [68]. Non-digestible fiber describes carbohydrates that pass through the small intestine intact, while the term prebiotics refers to a subset of non-digestible fiber that promotes microbial function. Within the category of non-digestible fiber, there are also distinctions between high-fermentable fiber, sometimes referred to as microbiota-accessible carbohydrates (MACs), and low-fermentable fiber. Each fiber subset interacts with gut microbiota in unique ways, with high-fermentable fiber contributing to microbe energetics and SCFA production and low-fermentable fibers promoting the efficacy of FMT treatments [21,70,72]. A comprehensive understanding of the relationship between fiber and the gut microbiome is still a work in progress, but fiber serves a clearly established critical role in providing substrates and energy necessary for a diverse and functional gut microbiome [68].

### 3.2. Short Chain Fatty Acids

SCFAs are the products of microbial fermentation of dietary fibers and resistant starches in the gut [73]. SCFAs are weak organic acids that differ in carbon chain length, with acetate being the most abundant, followed by propionate and butyrate. Acetate and propionate are predominantly produced by Gram-negative Bacteroidetes, while butyrate is primarily produced by Gram-positive Firmicutes [74]. SCFA production in the gut is approximately 35 µmol/kg body weight/hour [75] and can contribute up to 10% of human caloric requirements [76]. SCFAs are absorbed by the gut epithelium, where acetate and butyrate are rapidly metabolized into acetyl-CoA. Following absorption, propionate is subsequently transported to the liver and serves as a substrate for gluconeogenesis. Butyrate and propionate demonstrate the greatest potency of beneficial effects and play integral roles in metabolic regulation along the gut–liver axis, innate immunity, inflammatory response, and circadian rhythm. SCFAs exert their signaling effects through G-protein-coupled receptors (GPCRs) in colonic epithelial cells [74,77]. Propionate inhibits enzymes in the hepatic gluconeogenesis pathway through GPCR mediated signaling [78]. SCFA infusions increased secretion and upregulated genes involved in production of glucagon-like peptide-1 (GLP-1) in human cell lines, and increased fat oxidation, energy expenditure, plasma peptide YY (PYY) in human studies [74,79,80]. The effects of SCFAs on the secretion of GLP-1 and PYY are reported to occur through the FFAR2 GPCR signaling, although this specific mechanism requires further validation [81].

SCFAs are also associated with the amelioration of GI inflammatory markers through repression of TLR-activated mechanisms and by promoting mucus secretion and tight-junction integrity [82,83]. While all SCFAs demonstrate anti-inflammatory activity, the concentration threshold necessary for butyrate and propionate to induce these effects was significantly lower than acetate and lactate. SCFAs modulate the production of proinflammatory cytokine from TLR-activated intestinal epithelial cells, as well as secretion from TLR-activated macrophages, and reduce TLR-mediated CD40 upregulation [84]. SCFAs are also involved in transcriptional regulation through entrainment of systemic circadian rhythm, a process mediated through the inhibition of histone deacetylases (HDACs) [82,85,86,87]. Butyrate and propionate derived from microbial fiber fermentation have been shown to induce differentiation of both T-follicular regulatory cells in GALT and extrathymic Treg cells, as well as promote IL-22 production by CD4^+^ T cells, all mediated through GPR41/43 signaling for HDAC inhibition [44,88,89,90]. A recent study demonstrated that butyrate enhances hepatic lipid metabolism via the GPR41/43 pathway, suggesting that butyrate could be protective in the progression of metabolic dysfunction-associated fatty liver disease (MAFLD) induced by the Western-style diet [91].

### 3.3. Micronutrients

Colon-targeted delivery of vitamins such as vitamins B2 (riboflavin), B9 (folate), B12 (cobalamin), and K has shown potential in modulating gut microbial composition and functions [92]. Vitamin C supplementation has been strongly correlated with increased SCFA production and greater α-diversity within gut microbiota [92]. Vitamins C and E have both been linked to improved intestinal barrier integrity. To directly influence the microbiota population in the GI tract, these vitamins are often administered via colon-targeted formulations. In addition, vitamins A and D contribute to GI health by modulating immune responses and maintaining gut homeostasis [93]. One study comparing the gut microbial composition in children with autism spectrum disorder (ASD) before and after a single high dose of vitamin A showed a significant difference in the levels of *Bifidobacterium* [94], although the mechanism was unclear. In a South African study, vitamin A supplementation was associated with a reduction in bacterial diversity; however, paired studies have also reported that fungal (mycobiome) α-diversity increased under similar conditions, suggesting a differential impact on distinct microbial communities within the gut [95,96]. A recent study of 380 Cambodian schoolchildren found that vitamin A deficiency was linked to a distinct gut microbiota profile based on fecal samples, further underscoring its role in shaping microbial communities [97]. Vitamin D has both immune-modulating and anticancer properties, as vitamin D-activated genes are correlated with improved response to cancer treatments as well as increased survival. A study examining the potential of vitamin D as a determinant of cancer immunity and immunotherapy success found that it positively correlated with cancer resistance and a favorable gut profile rich in *Bacteroides fragilis* in mice [98].

Low vitamin B3 dietary intake in obese subjects has been correlated with reduced α-diversity and Bacteroidetes levels. One study examining the potential therapeutic effects of gut-targeted vitamin B3 supplementation showed positive effects on systemic insulin sensitivity and metabolic inflammation [99]. There is robust evidence for microbial synthesis pathways and the sharing of vitamin B with both the host and other commensals within the gut [100,101]. Some studies suggest that dysbiosis may result in vitamin B deficiencies, but microbial production of B12 is estimated to supply only about 2% of human needs [102].

Polyphenols are a diverse class of plant-derived micronutrients commonly found in fruits, vegetables, teas, chocolate, and other plant-based foods. Polyphenols provide protection from reactive oxygen species through their radical scavenging activity and are a subject of significant research activity widely studied due to their broad biochemical effects [103]. Beyond their well-established antioxidant and anti-inflammatory properties, polyphenols have demonstrated potential in treating non-communicable diseases, from Alzheimer’s to atherosclerosis to IBD [104]. These effects are thought to be mediated through microbiota-dependent pathways such as increasing SCFA production [105], downregulating inflammatory cytokines [106], improving gut barrier integrity [107], and shaping microbiota composition [108].

### 3.4. Diets (Western vs. Mediterranean, Animal vs. Plant Based)

The microbiome and its associated metabolic activities shift rapidly to adapt to changes in dietary profile. Diet-driven changes to the microbiome generally occur within 4 days of dietary intervention [22,66]. Interestingly, switching between animal-based and plant-based diets dictates changes in fundamental metabolic pathways. Metabolomic analysis of the gut microbiome under plant-based diets showed an upregulation of oxaloacetate (OAA) conversion to phosphoenolpyruvate (PEP), supporting de novo synthesis of aromatic amino acids. In contrast, microbial responses to animal-based diets promoted the reverse pathway and enhanced amino acid catabolism. An animal-based diet has been shown to increase the abundance of bile-tolerant microorganisms such as *Alistipes*, *Bilophila*, and *Bacteroides* while reducing populations of Firmicutes species involved in the fermentation of dietary plant polysaccharides, including *Roseburia*, *Eubacterium rectale*, and *Ruminococcus bromii* [22]. The fascinating ability of the microbiome to adapt to available nutrient profiles likely conferred a significant advantage on ancestors with inconsistent access to nutritional resources. In addition to modulating the microbiome through energy and nutrient availability, dietary intake can serve as an important source from which to introduce and maintain microbial diversity. Direct gut colonization by microbes derived from fermented foods has been demonstrated with regard to strains associated with cheeses and cured meats, such *Lactobacillus* and *Pediococcus* spp. [22,109]. There is enormous variability in the nutritional choices and dietary patterns on which humans subsist. Dietary patterns with high compositional diversity are highly correlated with microbiome stability, whereas consumption of a monotonous diet, such as meal-replacement beverages, is detrimental to microbiome stability [19].

The widely studied Mediterranean diet is characterized by whole grains, abundant fruits and vegetables, minimally processed foods, and lean proteins [110]. In studies examining the effects of a Mediterranean diet, adherence for the duration of one year was correlated with a decrease in the Firmicutes/Bacteroidetes ratio, along with an increased relative abundance of notably SCFA-producing strains such as *Dorea*, *Roseburia*, and *Coprococcus* [111]. The increase in SCFA-producing microbiota observed with the Mediterranean diet was not reproduced in a parallel group following the same diet under calorie restriction, signifying that energy deprivation alters the microbiome’s response to dietary interventions [111]. A Western-style diet, on the other hand, is characterized by greater consumption of animal protein, saturated fats, and sugars, correlated with lower microbiota abundance and decreases in SCFA production [112]. Several studies have shown that high-fat diets can disrupt the gut microbiome, often leading to reduced microbial diversity and enrichment of pro-inflammatory taxa [113,114]. However, it is important to note that not all high-fat diets exert the same effects; variations in fat type (e.g., saturated vs. unsaturated), source, and overall dietary composition can differentially shape the microbiome [115]. Administration of a Mediterranean diet enriched with olive oil over the course of one year led to a significant reduction in plasma BCAA levels [116]. BCAAs have been established as activators of nuclear factor kappa-light-chain-enhancer of activated B cells (NF-kB) mediated inflammatory signaling, as well as contributing to insulin resistance through activation of mammalian target of rapamycin (mTOR) complex-1 [117,118]. It is noteworthy that an animal-based diet resulted in a significant upregulation of bile salt hydrolases, enzymes that are required for the production of deoxycholic acid (DCA), which, in addition to causing increased liver cancer risk, suppresses the abundance of beneficial microbiota [22,119]. In addition, animal-based diets are rich in protein and saturated fat, which may cause the production of detrimental microbial metabolites, such as trimethylamine N-oxide (TMAO) and secondary bile acids, potentially promoting the risk of cardiovascular and liver diseases [120] (Figure 1).

Dietary patterns have a significant impact on metabolic function, chronic inflammation, immune system, and gut microbiota composition in human health. Western diets can promote the growth of microbiota enriched in pro-inflammatory bacteria and are associated with reduced microbial diversity and a shift toward less beneficial microbiome profiles [112,121]. This is further supported by findings from Vangay et al. (2018), which demonstrated that immigration to the U.S. and subsequent dietary Westernization led to significant loss of gut microbial diversity and increased abundance of Western-associated taxa [122]. In addition, the Western diet also promotes secondary-bile acid-producing bacteria such as *Bilophila wadsworthia* and *Alistipes,* which are associated with a high risk of metabolic syndrome and inflammation [121]. Importantly, Western-style diets high in saturated fats and simple sugars are associated with endotoxemia and elevated circulating levels of lipopolysaccharide (LPS), an endotoxin and a pro-inflammatory microbial molecule [123,124]. LPS can cross the intestinal barrier and stimulate inflammatory mediators in various tissues and organs via activation of the TLR pathway, which is present in most cells and macrophages. On the contrary, plant-derived diets support the growth of fiber-metabolizing bacteria and short-chain fatty acid (SCFA)-producing bacteria, including *Faecalibacterium prausnitzii* and *Roseburia*, which promotes anti-inflammatory effects and gut barrier integrity [121]. Plant-derived diets, which are generally high in polyphenols, omega-3 fatty acids, and fermented products, help mitigate inflammation and maintain mucosal immune homeostasis [125]. These results emphasize the significance of dietary approaches that prioritize diverse, fiber-rich, and marginally processed foods to improve resilient microbiome and reduce inflammatory and metabolic disease risk.

### 3.5. Ultra-Processed Foods

The Western diet is increasingly characterized by high consumption levels of ultra-processed foods (UPFs). The “ultra processed” classification within the NOVA system indicates the use of exclusively industrial processes to convert high-yield crops into components or products with extremely high longevity and palatability [126,127]. Examples of ultra-processed components include modified sugars, protein isolates, and hydrogenated oils, as well as emulsifying agents and synthetic color and flavor additives. Foods classified in the ultra-processed category include frozen and premade meals, reconstituted meat products, soft drinks, and packaged snacks and cookies. Studies estimate that approximately 60% of calories consumed by the US population were sourced from ultra-processed foods, with even higher consumption levels observed in younger and lower-income cohorts [128,129]. The ubiquitous presence of UPFs in modern diets prompts health concerns due to the additives present and nutrient profiles. 

Ultra-processed food consumption has been associated with a broad spectrum of adverse health outcomes. In a comprehensive meta-analysis, greater exposure to UPFs is linked with a higher risk of all-cause mortality, cardiovascular events, mental health outcomes, and metabolic disorders [130]. A large prospective cohort study spanning more than a decade found that UPF consumption level was positively associated with an increased risk of cancer and cardiometabolic disease [131]. Within this analysis, specific food subgroups such as sugar-sweetened beverages and animal-based products demonstrated the most significant associations with multimorbidity risk. The pronounced impact of UPF consumption on the gut microbiome composition suggests that UPF–microbiome interactions are a potential mechanism for disease pathogenesis [132,133]. Gut microbiome alterations mediated by UPF consumption have been implicated in the development of diabetic conditions [134], metabolic disorders [132], IBD [135,136,137], and allergic disease [138]. 

Accumulating evidence highlights the role of dietary additives commonly present in UPFs in disrupting gut microbiome function, potentially contributing to the pathogenesis of various diseases. A large prospective cohort study recently described significant associations between emulsifiers (e.g., carrageenan, sodium citrate, guar gum) and the risk of Type-2 diabetes [139]. Emulsifying agents can act similarly to non-ionic detergents, disrupting mucus barrier function and altering epithelial membrane permeability. For example, degradation products of carrageenan demonstrate pro-inflammatory properties that promote intestinal permeability and reduce insulin sensitivity in high BMI patients [140]. The emulsifying agent polysorbate-80 exhibits similar effects on gut permeability, decreasing the expression of proteins involved in promoting epithelial integrity and allowing for increased absorption of endocrine toxins [141]. In mouse models, polysorbate-80 administration facilitated the rapid onset of hepatic steatosis and significant increases in LPS, flagellin, and the intestinal inflammation marker serum lipocalin-2 [142]. Rather than affecting epithelial permeability, carboxymethylcellulose directly interacts with the human gut microbiome, promoting inflammatory capabilities and reducing both commensal abundance and SCFA production [143,144]. 

In addition to emulsifiers, common additives such as nanoparticles, colorants, and modified starches have been shown to disrupt the gut microbiome and are increasingly linked to disease development. Inorganic nanoparticle additives such as titanium dioxide and aluminosilicates can act as adjuvants to bacterial toxins to intensify inflammatory cytokine responses [145,146,147]. TiO_2_, an additive used to enhance white color in desserts and toothpaste, does not demonstrate direct host toxicity but has been shown to accumulate in commensal gut microbes, potentially altering microbial composition and SCFA production in mouse models [148,149]. These effects are more pronounced in obese mice, where TiO_2_ treatment resulted in distortion of intestinal epithelial structure, significant immune cell infiltration, increased levels of pro-inflammatory cytokine IL-12, and decreased levels of anti-inflammatory cytokine IL-10 [148]. The gut microbiome is also directly involved in the processing and absorption of synthetic colorants such as Red 40 and Yellow 6. The microbe-facilitated reduction in these additives contributes to the development of IBD in conditions of IL-23 expression [137]. Heavily modified carbohydrate additives have also demonstrated negative effects on gut functionality. Maltodextrin, a partially hydrolyzed starch used as a textural additive in a vast number of UPF products, can contribute to intestinal disease susceptibility by disrupting mucin production, inhibiting anti-bacterial cellular responses, and promoting the adhesion of pathogenic microbes [150,151,152]. In addition to emulsifiers, nanoparticles, and colorants, UPFs often contain antimicrobial preservatives to extend the shelf life of products, and exposure to these preservatives understandably affects gut microbial species. Chronic consumption of preservative compounds that contribute to dysbiosis by upregulating Proteobacteria species, inducing glucose intolerance, and significantly dysregulating microbial metabolite production [134]. 

Beyond their harmful additives, diets with elevated UPF content also promote dysbiosis and disease pathogenesis by contributing to nutrient and fiber deficiencies [153,154]. Diets high in UPFs are linked to increased caloric intake [155], often displacing potential health benefits provided by minimally processed foods. Generally possessing lower fiber content than minimally processed foods, UPFs are more efficiently absorbed and do not contribute the soluble fiber that is a crucial energy source for commensal microbes. UPF consumption alters microbiome composition and metabolite profiles, but further investigation is needed to directly implicate UPF consumption with decreased SCFA production in humans [133,156]. A recent randomized control trial studying fermentable carbohydrates versus maltodextrin highlighted the pervasive impact that UPFs can have on human health. Over the course of 5 weeks, administration of inulin-type fructans was associated with improved anxiety and depression indices when compared to maltodextrin-treated control groups [157]. These groups exhibited significant differences in gut microbiome composition, with upregulation of beneficial and SCFA-producing taxa such as *Bifidobacterium*, *Roseburia*, and *Faecalibacterium prausnitzii* in the fermentable fiber-treated group. These findings underscore the multifaceted impact of UPFs on the gut microbiome, highlighting how both harmful additives and nutrient-poor profiles may contribute to dysbiosis and disease development.

### 3.6. Probiotics and Antibiotics

Probiotics are defined as “Live microorganisms which when administered in adequate amounts, confer a health benefit on the host” [158]. It is important to note that not all probiotics are derived from fermented foods, and conversely, not all fermented foods contain live beneficial strains and thus are not necessarily considered probiotics. In population studies, without the ability to certify the health benefits of every microorganism-containing food, fermented food is often used as a placeholder for probiotic consumption. Individuals with fermented food-rich diets exhibit significant increases in microbial diversity, as well as decreases in several key cytokines and associated inflammatory factors, notably IL-6, IL-10, IL-12b, and TNF-α [71,159]. Direct gut colonization by microbes derived from fermented foods has been demonstrated with regard to strains associated with cheeses and cured meats, such *Lactobacillus* and *Pediococcus* spp. [22,109]. In some studies, the increased microbiota diversity was not reflective of the species present in the consumed probiotics, suggesting that probiotics may promote beneficial shifts in the gut environment that favor the emergence or growth of other commensal strains rather than acting solely through direct colonization [71]. This could be a result of differential receptivity to microbiome colonization. Upon reaching the gut, probiotics encounter significant mucosal resistance from the existing microbiome, which results in highly individualized colonization patterns [160]. There can be significant differences in the reported success of probiotic colonization based on whether fecal or mucosal sampling methods were used, as dietary strains can still be detected as washout in the feces following failure to colonize the gut [160].

The mechanisms of probiotic benefit are typically related to supporting microbial diversity and stability, shifting the microbiome towards a beneficial gut metabolite profile. The metabolomic analysis found significant increases in conjugated linoleic acid (CLA) abundance in fecal samples of fermented food consumers [109]. CLA is found in high abundance in ruminant animal products due to the high abundance of species with fatty-acid conjugation activity in the ruminant gut microbiome [161]. Strains of *Lactobacillus, Bifidobacterium,* and *Roseburia,* among others found in the human gut microbiome, have demonstrated CLA production activity [162,163]. Multiple studies suggest that endogenous production of CLA does not reach sufficient levels to exert its beneficial effects beyond the environment in which it was produced [162,164]. CLA has been proposed to have anti-tumorigenic effects by promoting the accumulation of p53 and subsequent cell growth arrest [165]. D-phenyllactic acid (D-PLA), a metabolite of lactic acid bacteria (LAB) that is highly enriched in LAB-fermented foods, is a potent agonist of a G-protein coupled receptor of hydroxycarboxylic acid (HCA3) that plays an important role in neutrophil chemotaxis and adipocyte lipolysis regulation [166,167]. The highest levels of HCA3 expression in the body are found in neutrophils and monocytes, and increased plasma concentrations of D-PLA following consumption of LAB-fermented foods were sufficient to activate the chemotactic response mediated by HCA3 [168].

There is evidence of the efficacy of probiotic administration in the treatment of GI disorders: infectious and acute diarrhea, ulcerative and necrotizing colitis, and irritable bowel syndrome, among other GI disorders [169]. The beneficial effects of probiotics on inflammatory response and general health have implications along the gut–brain axis and have shown potential in the treatment of multiple sclerosis, as well as reported improvements in depression, generalized anxiety, and stress [170,171,172]. Administration of *Lactobacillus* spp. improves intestinal epithelial integrity and renal function in murine lupus models [173]. Probiotic treatment induced shifts toward Th2 type response, along with increases in anti-inflammatory cytokines (IL-4 and IL-10) and decreases in pro-inflammatory mediators, like IFN-y, TNF-α, and IL-17 [174,175]. In a study by Lavasani et al., a novel probiotic mixture shows significant therapeutic potential in ameliorating experimental autoimmune encephalomyelitis (EAE), primarily through the induction of IL-10-producing regulatory T cells, which underscores the immunomodulatory effects of probiotics in autoimmune disorders [175]. The VSL#3 strain probiotic, the only probiotic currently classified as a medical food, has demonstrated the capability to increase butyrate production and GLP-1 secretion, thereby protecting against diet-induced obesity and insulin hypersensitivity [79,176].

Given the broad scope of benefits associated with cultivating the microbiome with probiotics, antibiotics have understandably been studied for their detrimental effects on microbiome composition and function. Antibiotic suppression of the gut microbiome has been implicated with wiping out circadian transcription patterns, as well as conferring risk for IBD, celiac disease (CeD), and bacterial pneumonia, among others [16,177,178]. A significant concern with the use of antibiotics is the emergence of antibiotic-resistance genes (ARG). Antibiotic treatment increases the number of antibiotic resistance genes detected in the lower GI tract. The effects of probiotic administration are dependent on receptivity to colonization. In colonization-resistant individuals, probiotic follow-up to an antibiotic course expands the quantity and distribution of ARG expression [179]. Following antibiotic-induced disruption, probiotic administration was shown to be detrimental to the reconstitution of a homeostatic microbiome. Introduced into a dysbiotic environment following antibiotic treatment, common probiotic strains such as *Lactobacillus* can become dominant and secrete factors inhibiting a return to balanced microbiome composition [180]. Conversely, another notable consequence of antibiotic treatment is the increased availability of host-derived free sialic acid in the gut, which is the nutrient source of opportunistic pathogens like *Salmonella typhimurium* and *Clostridium difficile*, enhancing their abundance in the gut and potentially leading to infections [181].

### 3.7. Dietary Cycle and Circadian Rhythms

In mammals, the suprachiasmatic nucleus (SCN) is responsible for coordinating circadian rhythm throughout the body [182]. Intrinsically photosensitive retinal ganglion cells carry light stimulus information from the environment to the retinohypothalamic tract, which carries an action potential down to the SCN. The SCN then synapses with the paraventricular nucleus, which is responsible for the synchronization of peripheral systems, and eventually, the pineal gland, which then releases melatonin into the bloodstream [182]. At a cellular level, rhythmicity is facilitated by families of genes that encode activation factors (CLOCK, BMAL1) or repression factors (PER1, PER2, CRY2), which form an autoregulatory feedback loop and drive diurnal transcription patterns [183,184]. The activation and regulation of these transcription factors is dependent on tissue type and organ system. While stimulatory input from the light/dark cycle is a primary driver of circadian rhythmicity due to its direct integration with the SCN, facilitation of this rhythmicity throughout peripheral tissues is a balance of environmental and internal stimuli [185]. For example, peripheral clock mechanisms are highly sensitive to temperature fluctuations as an entraining factor, physical activity is a driver of circadian genes in skeletal muscle, and various modes of hormone signaling have been shown to facilitate rhythmicity in the cardiovascular, gastrointestinal, and hepatic organ systems [186,187,188,189,190]. Each organ is subject to this balancing between SCN and environmental inputs, with tissues such as the liver, kidneys, and GI tract exhibiting susceptibility to modulation by the latter [191].

The peripheral clocks of the GI, liver, and kidneys have demonstrated significant entrainment by temporal feeding patterns and alterations to the gut microbiome. Mice devoid of functional cellular clock machinery genes showed little to no oscillation in bacterial abundance and adherence to intestinal mucosa. Diurnal fluctuations of Bacteroidetes and Firmicutes abundance are erased in clock-gene deficient mice, but imposing a restricted light-phase-only feeding pattern was sufficient to retrain the rhythmicity of microbiota abundance [192]. Similarly, imposing a dark phase-only feeding pattern on wild-type mice inverted the localization patterns of the microbiome, implying a pivotal role for dietary patterns in driving the abundance and biogeographical localization of microbiota [16].

Patterns of rhythmic localization of bacterial metabolites not only influence but actively drive gene expression in both the large intestine and the liver. Mice treated with broad-spectrum antibiotics lost oscillations in transcription levels of genes involved in nucleotide metabolism and cell cycle compared to their control group counterparts. The impact of the microbiome on transcriptional regulation was determined to be dependent on adherence to the mucosal membrane, not simply presence in the region, further cementing the importance of biogeographical localization patterns [16]. The microbial metabolome influences rhythmic host physiology beyond the intestine by driving systemic oscillations at metabolite levels and shaping transcriptional rhythms in the liver by polyamine signaling [16]. Gut microbiome-mediated oscillations in hepatic transcription have potentially significant implications in the diurnal rhythmicity drug metabolism [193,194]. Studies have demonstrated that central-clock deficient and germ-free strains, as well as groups administered antibiotic treatments, did not exhibit fluctuations in hepatotoxicity markers following drug administration [16].

Diet and microbiome-mediated circadian rhythmicity have a bidirectional relationship that interacts with many variables, not just feeding schedules. Studies have shown that jetlag induces a dysbiotic state characterized by increased glucose intolerance and induced obesity in a manner that is transferable by fecal microbiota transplantation (FMT) [195]. Similarly, inducing obesity via a Western-style diet disrupted the normal diurnal fluctuations in whole-body metabolism, along with altered expression of core clock genes in the hippocampus [196]. On the other hand, microbiome-mediated mechanisms, such as butyrate production, maintain rhythmicity through upregulation of clock genes and improve quality of sleep in ulcerative colitis (UC) patients [82]. In our recent study in Dahl salt-sensitive (S) rats, we demonstrated that diurnal shifts in gut microbial composition, i.e., the ratio of Firmicutes/Bacteroidetes abundance (F/B), are closely associated with the development of salt-sensitive hypertension and kidney injury [197]. Thus, the interplay between diet, gut microbiome, and rhythmicity allows for diversity and resilient gut microbiome to provide a measure of protection from circadian disruption and associated consequences [198,199].

## 4. Diet–Microbiota Interactions in Disease Pathogenesis

### 4.1. Cardiovascular Diseases

Gut dysbiosis, or the imbalance of the composition and function of the gut microbiota, has become increasingly recognized as a major contributor to the pathogenesis of many key manifestations of cardiovascular disease (CVD), including hypertension, atherosclerosis, and heart failure [200]. This occurs through the induction of chronic, low-grade inflammation due to a compromised intestinal barrier, creating a “leaky gut” effect that allows the systemic entry of pro-inflammatory molecules like lipopolysaccharide (LPS) and other harmful microbial products [201]. In time, the inflammatory effects of these products contribute significantly to both vascular disease and cardiac failure and thus are of great interest in understanding these morbidities in connection to gut health [202,203,204].

Furthermore, gut dysbiosis promotes the generation of specific co-metabolites such as trimethylamine-N-oxide (TMAO), which also contributes to this low-grade inflammatory effect via several mechanisms and is intricately tied to the gut microbial composition [201]. TMAO is generated by the process of oxidation of trimethylamine (TMA) by the hepatic flavin monooxygenases (FMO1 and FMO3) via bacterial degradation of dietary carnitine, choline, and lecithin found primarily in meat and eggs, establishing a specific link between diet and CVD. Serum levels of TMAO have been correlated with greater risk of atherosclerosis and coronary artery disease and may serve as a useful biomarker for assessing the overall risk of adverse cardiovascular events [205]. Previous mouse studies have shown that a choline-rich diet with an intact gut microbiome leads to increased TMAO and atherosclerotic plaques, while mice with a compromised microbiome did not show these effects under similar conditions [206] (Figure 1). While elevated TMAO levels have been associated with gut dysbiosis and cardiometabolic disorders, the direction and mechanism of this relationship remain debated. Landfald et al. (2017) suggest that TMAO may not directly alter microbiota composition but could indirectly promote dysbiosis by supporting the growth of TMA-producing microbial populations under certain conditions [207].

Short-chain fatty acids (SCFAs) such as propionate and butyrate are well-studied for their protective anti-inflammatory effects on the cardiovascular system [208]. SCFAs are formed via anaerobic fermentation and, therefore, promoted by diets rich in fiber, a component often lacking in Western diets [200]. SCFAs exert their protective effects via numerous mechanisms, such as butyrate’s inhibitory effect on histone deacetylases (HDACs), which curtails the production of pro-inflammatory cytokines [208] (Figure 1). Conversely, dysbiosis alters the bile acid profile in the GI tract, which can disrupt signaling via the farnesoid X receptor (FXR) and Takeda G protein-coupled receptor 5 (TGR5) and ultimately impair lipid metabolism, another important factor in CVD pathogenesis [200]. The complex interplay of various key molecules and the disharmony of gut microbes belies the complexity of this relationship between the gut and heart. Understanding these connections may provide new avenues for the development of novel therapeutics targeting highly relevant conditions such as hypertension and atherosclerosis.

The specific dietary underpinnings of gut dysbiosis as it relates to CVD are not yet fully understood [209]. However, its intimate link with diet has long been known, as seen with the extensively studied Mediterranean diet and its many remarkable preventative benefits [209]. Microbial communities are continually shaped by dietary inputs, and identifying the most important factors shows promise in providing many new answers to our health. Limited studies have already shown that a highly targeted diet can not only halt but even reverse atherosclerosis under the right conditions [210]. Cardiovascular disease remains the single leading cause of death and disability in the United States, and a better understanding of how gut health directly affects cardiovascular function may provide new paradigms for combating CVD [206,209].

### 4.2. Obesity

Obesity is a disease of global incidence and diverse etiology, but several commonalities emerge upon consideration of the gut microbiome. In attempts to characterize the gut microbiome of obese individuals, a large body of literature highlights compositional metrics, such as the ratio between Firmicutes and Bacteroidetes phyla abundance. Obesity strongly correlates with increases in F/B ratio and Proteobacteria abundance, alongside notable reductions in *Akkermansia muciniphila* and *Faecalibacterium prausnitzii* abundance [211,212,213]. This compositional identity is directly shaped by consumption patterns, with Firmicutes demonstrating a competitive advantage in high-energy-density diets [56]. For instance, high-energy-density diets, such as those rich in ultra-processed foods, induce changes in the gut microbiome composition associated with increased leptin levels and the development of leptin resistance. Impaired leptin signaling can inhibit feelings of fullness, thereby promoting overconsumption and increasing obesity risk [132]. The microbiome of obese individuals is further distinguished by several functional differences. At a fundamental level, greater energy harvest predisposes individuals to obesity and metabolic disorders, whether driven by high-calorie diets or enhanced efficiency in extracting energy from food. The gut microbiome contributes to obesity through a positive feedback loop involving the latter mechanism, as the compositional changes following induced obesity result in a greater capacity to harvest energy from the diet [214]. This efficiency advantage in gleaning energy from the diet is transferable through the maternal transfer of microbiota and FMT, solidifying the microbial mediation of this effect and underlining the importance of addressing obesity within the context of the gut microbiome [215,216].

Microbial metabolites show a high degree of involvement in the mechanisms of obesity. For example, rats maintained on a high-fat diet (HFD) exhibit chronic upregulation of microbial acetate production, which activates the parasympathetic nervous system and increases ghrelin and glucose-stimulated insulin secretion [217]. These secretions induce elevated hunger and promote energy storage as fat, as well as playing into a positive feedback loop that sustains higher consumption levels. Chronic activation of this mechanism promotes obesity, hyperlipidemia, fatty liver disease, and insulin resistance. Indeed, acetate production is upregulated following the consumption of calorically dense food, suggesting an evolutionary mechanism where acetate production induces elevated hunger response and energy storage to capitalize on a high-density food source [217].

Despite the role of acetate in promoting hyperphagia and hyperlipidemia, SCFAs have generally demonstrated beneficial metabolic effects. Colonic SCFA infusion increases fasting fat oxidation, resting energy expenditure, and plasma peptide YY (PYY) while decreasing fasting free glycerol concentrations [217]. Butyrate has been shown to exert protective effects against insulin resistance and obesity by promoting fatty acid oxidation and enhancing adaptive thermogenesis. Morphologically, these effects are marked by an upregulation of mitochondrial function and biogenesis in skeletal muscle and brown fat. Mechanistically, butyrate’s HDAC inhibitory activity is thought to facilitate increases in PGC1-α and peroxisome proliferator-activated receptor (PPAR)-δ, which promotes fatty acid oxidation in skeletal muscle [218]. Dietary SCFAs similarly stimulate oxidative metabolism by downregulating PPAR-γ expression, activating mitochondrial uncoupling proteins, and increasing the AMP-ATP ratio. At a systemic level, SCFA supplementation facilitated reductions in body weight and hepatic steatosis while improving insulin sensitivity, all in a PPAR-γ dependent manner [219].

Other dietary interventions have shown potential to combat obesity through the modulation of gut microbiota. Across various microbiome-targeted obesity treatment strategies, dietary fiber intake appears to be an effective adjunct therapy. Dietary supplementation with low-fermentable fiber enhanced the ability of FMT to improve insulin sensitivity in patients with concomitant obesity and metabolic disorders [21]. High fermentable fiber supplementation notably decreased levels of pro-inflammatory bacterial metabolites such as TMAO and indoxyl sulfate (IxS) [220,221]. Diets high in resistant carbohydrates prompted a significant reduction in body weight and BMI in both diet-induced and genetic obesity [67,221]. High-protein calorie restriction diets can improve obesity by inducing energetic conditions that pressure the gut microbiome into compositional shifts and increase diversity [61]. It is reported that the introduction of CLA into the patient diet has been associated with reductions in F/B ratio and visceral fat mass and increases in SCFA concentrations and the abundance of Bacteroidetes genera involved with downregulation of lipid metabolism and hepatic steatosis [222]. Tea-derived polyphenols similarly correlated with increases in SCFA-producing species, accompanied by compositional shifts marked by a significant reduction in F/B ratio and upregulated expression of genes related to carbon metabolism [108].

Certain bacterial strains have been isolated and studied for mechanisms that confer protective effects against obesity. In murine models of obesity, HFD promotes NF-kB activation and suppresses AMP-activated protein kinase (AMPK) and SIRT1 expression [223]. NF-kB facilitates increased inflammatory activation, and AMPK and SIRT1 are essential in facilitating energy homeostasis and fat oxidation. *Lactobacillus sakei* administration has been shown to counteract the effects of HFD by helping to normalize NF-kB, AMPK, and SIRT1 expression [223]. Similarly, *Bifidobacterium adolescentis*, *Lactobacillus mucosae*, and *Weissella cibaria* have been shown to induce the expression of IL-10, a potent anti-inflammatory cytokine. Oral gavage of these species abrogated HFD-induced obesity, colitis, and hepatic steatosis in mice [224]. In addition to diet-induced obesity causing upregulating metabolic dysregulation and systemic inflammation, core clock genes in the hippocampus were disrupted after high-sugar, high-fat feeding [196]. This further emphasizes the diffuse influence of the gut microbiome on obesity through central mechanisms of behavioral and neural control.

### 4.3. Type 1 Diabetes

Type 1 diabetes (T1D), a multifactorial disease marked by the presence of islet auto-antibodies, is significantly influenced by disruptions within the intestinal microbial community and its subsequent impact on host immunity. “Leaky gut” can provoke excessive inflammatory responses and the over-activation of T-cells, which may then migrate to the pancreatic lymph nodes, contributing to the autoimmune assault that characterizes T1D [225,226]. Landmark initiatives such as The Environmental Determinants of Diabetes in the Young (TEDDY) study have been instrumental in characterizing the early gut microbiome. Findings from TEDDY and other metagenomic studies indicate that the microbiomes of healthy individuals often possess a greater abundance of genes related to fermentation and the biosynthesis of short-chain fatty acids (SCFAs) [227]. Conversely, a reduction in SCFA-producing bacteria, particularly those yielding butyrate, has been linked to T1D [228]. SCFAs, especially butyrate, are thought to confer protective effects against early-onset T1D. One proposed mechanism for this protection involves the maintenance of epithelial barrier function [229]. Butyrate administration, for instance, has been shown to improve insulin resistance and fortify the intestinal barrier by promoting the secretion of glucagon-like peptide-1 (GLP-1). GLP-1, in turn, enhances the production of colonic mucin and tight junction proteins, thereby strengthening gut integrity [230]. Intake levels of total dietary fiber, fermentable fiber, and available carbohydrates have demonstrated a positive correlation with fecal SCFA concentrations in T1D patients. This highlights a modifiable avenue through which beneficial microbial shifts and metabolite production can be encouraged [231].

Therapeutic strategies aimed at modulating the gut microbiota are areas of active investigation, though they also underscore the complexities involved. A recent randomized controlled trial exploring fecal microbiota transplantation (FMT) yielded intriguing results: autologous FMT (using the patient’s own stool) was found to be more effective in preserving insulin production than FMT from healthy donors. This unexpected outcome reveals significant gaps in our current understanding of the intricate mechanisms connecting the gut microbiota to T1D pathogenesis and host compatibility in microbial therapies [232]. In contrast, probiotic supplementation is emerging as a potentially more straightforward adjuvant therapy. Building on the knowledge of specific probiotic strains linked to host immune regulation, a randomized, double-blind, placebo-controlled trial demonstrated that probiotic supplementation alongside standard insulin therapy in T1D patients led to reductions in hemoglobin A1c (HbA1c) levels and more stable glycemic control compared to insulin therapy alone. These findings suggest that probiotics could offer a valuable advancement in the standard of care for T1D by favorably modulating the gut-immune dialogue [233].

### 4.4. Type 2 Diabetes

The gut microbiome plays a critical role in the pathophysiology of type 2 diabetes (T2D). Consistent gut alterations are observed in individuals with T2D, including a marked reduction in butyrate-producing bacteria and an increase in opportunistic pathogens [234]. Diabetic patients also show decreased levels of Firmicutes and Clostridia, while ratios such as F/B and *Bacteroides*/*Prevotella* to *C. coccoides*/*E. rectale* increases proportionally with plasma glucose levels [235,236]. Such microbial shifts are associated with systemic metabolic disruption, inflammation, and impaired glycemic control.

Microbial metabolites such as SCFAs play an important role in modulating host glucose metabolism. Higher circulating levels of SCFAs have been associated with increased insulin sensitivity and elevated fasting GLP-1 levels [237]. Genetic data suggest that enhanced endogenous production of butyrate and acetate is beneficial for cellular insulin response, and dysregulation of propionate production or absorption increases the risk of developing T2D [238]. Not all butyrate-producing species are uniformly beneficial, however, and some have been correlated with dysglycemia, suggesting a need for more nuanced characterization [239].

Diet modulates microbiota composition in clinically meaningful ways. Preservative agents found in ultra-processed foods have been found to induce glucose intolerance by dysregulating microbial amino acid synthesis, contributing to reductions in GLP-1 release [134]. On the other hand, high-fiber, polyphenol-rich diets increase microbial α-diversity and favor the proliferation of beneficial taxa such as the SCFA-producing *Faecalibacterium prausnitzii* and *Akkermansia muciniphila* [240,241]. Prebiotic fiber interventions have been shown to reduce HbA1c levels and diabetes-related distress, with these benefits linked to an increased abundance of SCFA-producing gut microbes [242,243,244]. Dietary interventions with high amylose starch content have also been shown to increase SCFA production, improve glycemic outcomes, and promote butyrate-producing species [245,246]. The use of probiotic supplementation to modify the gut microbiome has shown promise in improving glycemic control. In a six-week trial, probiotic administration corresponded with reduced fructosamine and HbA1c levels in diabetic patients [159]. Meta-analyses of probiotic studies have consistently shown reductions in fasting blood glucose, although effects on HbA1c are less consistent due to variations in patient populations and study designs [247,248,249,250,251,252].

Microbial dysbiosis has also been implicated in T2D-related peripheral complications. Patients with diabetic retinopathy exhibit unique microbial signatures marked by a loss of SCFA-producing and anti-inflammatory species [253]. This pattern suggests that a disease-altered microbiome profile contributes to such complications via a loss of anti-inflammatory signaling as opposed to an increase in pro-inflammatory signaling. Reintroducing beneficial taxa via FMT has demonstrated improvements in insulin resistance and peripheral symptoms (e.g., distal polyneuropathy) through mechanisms that promote butyrate production and dampen the production of inflammatory cytokines [254,255,256]. Pharmacological treatments further complicate the interaction between microbiome and host metabolism. Metformin, a common T2D medication, has been shown to reduce microbial diversity while enhancing bile acid metabolism and FXR signaling, which may contribute to its therapeutic effects [257,258,259]. However, it remains unclear whether these microbiome changes are driven by the drug or by improved metabolic status [260]. Acarbose, an α-glucosidase inhibitor, similarly affects microbiome composition, increasing SCFA production in a diet-dependent manner [246].

The integrity of the gut barrier is frequently compromised in T2D and is further weakened by low dietary fiber intake. In fiber-deficient states, microbes resort to degrading the gut’s mucosal barrier by consuming host-derived mucus proteins, leading to increased intestinal permeability [261,262]. SCFAs can counteract this effect by stimulating MUC2 production in goblet cells, thereby enhancing mucosal protection [263]. The influence of the microbiome on host metabolism extends beyond SCFA production. Emerging evidence suggests that gut-derived serotonin plays an important role in glucose regulation, with inhibition of peripheral serotonin synthesis, either through genetic manipulation or antibiotic treatment, which results in improved glucose clearance and enhanced insulin sensitivity [264,265]. These findings highlight the shared pathway linking microbial activity, serotonin metabolism, and glycemic control. Lifestyle interventions, such as exercise, also influence the gut microbial composition, which may exert T2D outcomes. For example, moderate-intensity continuous exercise has been shown to increase the abundance of butyrate-producing species more effectively than high-intensity interval training [266]. In addition, supplementation with *Akkermansia muciniphila* has been associated with improved insulin sensitivity and reductions in liver inflammation and dysfunction [267], underscoring the potential for non-pharmacologic, microbiome-targeted therapies in comprehensive T2D management. Diets high in saturated fatty acids, such as palmitate, demonstrate an ability to impair pancreatic β cell function via TLR4/MyD88 pathway activation and monocyte recruitment [268]. The subsequent recruitment of M1-type proinflammatory monocytes/macrophages thereby contributes to inflammation, insulin resistance, and ultimately β-cell dysfunction [268]. These results highlight the importance of dietary lipids in modulating components of the immune system, which play a key role in the pathogenesis of T2D.

### 4.5. Respiratory Disease

The gut microbiome plays a critical regulatory role in shaping immune responses to respiratory infections, and gut dysbiosis leads to worsened disease outcomes. In murine models, depletion of the gut microbiome prior to *Streptococcus pneumoniae* infection heightened inflammation, organ damage, and mortality, largely due to altered alveolar macrophage activity and reduced immune function [269]. This defective pulmonary immune response is associated with IL-10 upregulation, observed in germ-free mice following respiratory infection [269,270]. It has also been demonstrated that this loss of immune function can be rescued via TLR agonists, anti-IL-10 antibodies, or conventionalization of the gut microbiome, further supporting this proposed mechanism [270]. Antibiotic-induced dysbiosis also disrupts the production of APRIL, a TLR-dependent factor essential for IgA class-switching, leading to IgA deficiency and increased vulnerability to pathogens such as *Pseudomonas aeruginosa* [30,178].

Much as gut dysbiosis negatively impacts respiratory tract infections, such infections can conversely lead to potentially harmful alterations in the gut microbiome. *Mycobacterium tuberculosis* (MTB) infection has also been shown to significantly affect microbiome composition in both the gut and lungs. Studies indicate that tuberculosis (TB) patients often exhibit reduced microbial diversity and disrupted microbiota during active disease and throughout the course of treatment [271,272]. These alterations may impact disease progression, immune regulation, and even treatment outcomes. Emerging evidence suggests that MTB infection can lead to gut microbiome shifts through mechanisms involving systemic immune signaling and inflammatory responses [271]. Co-infection with *Helicobacter* species, for example, has been associated with altered susceptibility to MTB infection, while oral anaerobes that translocate into the lung may produce metabolites that impair pulmonary immunity and predict disease progression [272].

Moreover, prior TB treatment is believed to deplete T-cell epitopes on commensal non-tuberculous *Mycobacteria*, increasing susceptibility to reinfection [271]. Prolonged antibiotic therapy, an essential component of TB treatment, has also been shown to exert lasting effects on both the gut and lung microbiota, influencing immune recovery, microbiome resilience, and possibly relapse risk [271,272]. Incorporating TB into the broader framework of respiratory microbiome dynamics underscores the importance of a holistic understanding of microbial contributions to pulmonary health and disease, especially within high-burden regions [272].

COVID-19 patients with high fecal viral loads show enhanced nucleotide and amino acid metabolism, while those with low viral deposition display increased SCFA-producing bacteria [273,274]. These shifts are partly driven by IFN-1 signaling, which disrupts anti-inflammatory pathways in the gut [275]. Respiratory viruses like COVID-19 and influenza also often cause gastrointestinal symptoms as a consequence of lung-derived immune signaling. Specifically, CD4^+^ cells migrate to the gut via CCL25/CCR9 and secrete IFN-γ, driving Th17 lineage polarization and contributing to tissue dysfunction [276]. Many such mechanisms connecting gut health to the pulmonary system have similarly been elucidated.

Respiratory infections also elevate IL-22 secretion, upregulating RegIIIγ (antibacterial lectin) and altering the microbiota composition [277]. Moreover, gut microbiota influences IL-22 levels through the production of aryl hydrocarbon receptor (AhR) ligands derived from dietary tryptophan [278,279]. Tryptophan deprivation has been shown to increase TReg populations and potentially improve immune functioning, though its therapeutic potential in chronic infection remains unclear [280]. In addition, high-fiber diets can confer early resistance to respiratory viruses through SCFA-mediated GPR41/GPR43 activation and induction of IFN-1-regulated gene expression in lung stromal cells, highlighting a diet–microbiome–immune axis connection to respiratory health [281,282]. Activation of GPR41 also upregulates the release of Ly6c^−^-patrolling monocytes from the bone marrow. These monocytes exert anti-inflammatory effects and oversee tissue repair in severe influenza infection [283]. The influence of dietary metabolites on interferon signaling and monocyte recruitment highlights a microbiome–immune axis crucial for respiratory health.

In the context of chronic obstructive pulmonary disease (COPD), the gut microbiome may play as both a contributor and a potential therapeutic target. COPD is associated with gut dysbiosis, characterized by *Prevotella* dominance and significantly reduced SCFA levels [284]. FMT from healthy donors has been shown to improve markers of COPD-associated inflammation, alveolar destruction, and lung function. In contrast, FMT from COPD patients or mice exposed to high levels of cigarette smoke-induced lung inflammation in recipient mice [284,285]. Dietary interventions and resistant starch diets alleviate airway inflammation and alveolar destruction caused by cigarette smoke exposure by enhancing microbial SCFA production [285]. Similarly, dietary inulin supplementation in human COPD patients correlated with improved COPD Assessment Test scores, accompanied by gut microbiome compositional shifts favoring increased Bacteroidetes abundance [285]. Notably, acute COPD exacerbations were associated with decreased relative abundances of Firmicutes and Actinobacteria, along with increased Bacteroidetes abundance [286]. Active chronic smokers exhibit distinct gut microbiome profiles, marked by increased Bacteroidetes and decreased Firmicutes and Proteobacteria abundance [287]. Correlational studies between specific taxa and reduced lung function suggest *Streptococcus* or *Lachnospiracae* may serve as potential biomarkers of COPD [288].

### 4.6. Chronic Kidney Disease

The primary mechanism through which the gut microbiome interacts with kidney pathogenesis is through the production of protein-bound uremic toxins. Compounds such as indoxyl sulfate (IxS) and p-cresol sulfate (pCS) are produced via the metabolism of tryptophan and aromatic amino acids by gut microbes. Furthermore, in healthy individuals, uremic toxins are effectively cleared by proximal tubule organic anion transporters; however, as renal function declines, these toxins accumulate and contribute to the progression of chronic kidney disease (CKD) [289,290].

Impaired kidney function and subsequent retention of waste products prompt an upregulation in the proteolytic bacteria responsible for the production of uremic toxins [291]. IxS and pCS contribute to the progression of chronic kidney disease (CKD) by inducing NADPH-mediated production of reactive oxygen species (ROS), inhibiting antioxidative and superoxide scavenging functions in renal tissues [292,293,294]. The oxidative stress induced by IxS and pCS has a secondary effect of promoting robust activation of the renin-angiotensin-aldosterone system (RAAS). In addition to inducing damage by increasing glomerular hydrostatic pressure, RAAS signaling promotes fibrosis via upregulation of TGF-β/Smad pathway proteins, fibronectin, and α-smooth muscle cell expression [295].

Given the integral role that the gut microbiome plays in the production of uremic toxins, modulation of GM composition and function have therapeutic potential in the treatment of CKD. Dietary fiber supplementation has shown a robust ability to facilitate reductions in plasma uremic toxin levels [296,297,298]. There are multiple proposed mechanisms by which dietary fiber could mediate this effect. Dietary fiber has a well-established role in supporting the function of butyrate-producing bacteria. Therefore, increasing fiber consumption promotes intestinal epithelial integrity and inhibits leakage of uremic toxins. In longitudinal studies, patients who went on to develop CKD demonstrated a lower relative abundance of butyrate-producing microbes [299]. Alternatively, the abundance of fiber decreases microbe reliance on protein metabolism and reduces the production of uremic toxin precursor molecules [300]. This hypothesis has been supported in cross-sectional patient trials, with higher dietary fiber and plant-based diets corresponding to lower levels of IxS and pCS, respectively [301]. Conversely, patients on high-protein diets demonstrate higher plasma IxS levels [302,303].

Shaping gut microbiota composition and function through oral supplementation, such as probiotics and prebiotics, has shown variable results. A clinical study by *Borges* et al. showed that probiotic administration had no significant effect on gut microbiota composition or inflammatory markers and even facilitated increased uremic toxin levels in CKD patients undergoing dialysis treatment [304]. Conversely, oral FMT supplementation from healthy individuals stabilized renal function parameters and slowed disease progression regardless of clinical stage by reducing serum creatinine and uremic toxin levels [305]. Furthermore, individuals with diabetes and hypertension are at high risk for developing CKD, but treatment with oral FMT has been associated with a significantly reduced rate of CKD progression [305], highlighting the therapeutic potential of gut microbiota in high-risk populations.

### 4.7. Chronic Liver Disease

Emerging evidence underscores the influential role of the gut microbiome in pathogenesis and potential treatment of chronic liver diseases, including MAFLD. In a four-month placebo-controlled study, a resistant starch (RS) diet significantly reduced intrahepatic triglyceride content and liver injury markers in MAFLD patients, alongside reductions in liver injury markers [67]. Metagenomic analysis revealed a strong positive correlation between the abundance of *Bacteroides stercoris* and levels of both intrahepatic triglycerides and plasma markers of liver injury, suggesting a mechanistic link. This association was further validated through FMT, where the transfer of microbiota from RS-treated donors significantly reduced hepatic steatosis, ALT and AST levels, hepatic triglycerides, and the expression of hepatic genes involved in inflammation and immune cell recruitment [67]. The pathogenic role of *B. stercoris* in MAFLD has been further confirmed by oral administration studies, where the strain nearly doubled intrahepatic triglyceride levels.

Conversely, depletion of beneficial taxa such as *Ruminococcus*, *Coprococcus*, *Akkermansia muciniphila*, and *Faecalibacterium prausnitzii* was consistently associated with MAFLD in biopsy-confirmed adults [18], although taxonomic findings across studies remain mixed. For instance, some studies associated *Ruminococcus* with cirrhosis and *Bacteroides* with increasing MAFLD severity [306], while others failed to confirm consistent taxonomic correlations. A cross-sectional Taiwanese study reported a reduced F/B ratio in MAFLD along with decreased Clostridia and *Ruminococcaceae*, contrasting European findings such as those by Boursier et al. [306,307]. Diverse microbiome datasets from both Chinese and European cohorts consistently reported elevated *B. stercoris* levels in MAFLD patients compared to controls, reinforcing its potential role as a microbial contributor to disease progression [308,309].

Alterations in the gut microbiota composition play a direct role in the pathogenesis of liver diseases, including alcohol-associated liver disease (ALD), metabolic dysfunction-associated steatohepatitis (MASH), and hepatic encephalopathy. *Candida albicans*, which are found in higher abundance in the gut of ALD patients, produce the endotoxin candidalysin, which directly damages hepatocytes and exacerbates alcohol-associated liver injury and steatosis [310]. Disulfiram, a drug used in the clinical treatment of chronic alcoholism, suppresses *Clostridium* abundance, functionally inhibiting secondary bile acid biosynthesis and mitigating liver inflammation and fibrosis in MASH models [311].

Similarly, the gut-targeted antibiotic Rifaximin-α has been shown to prevent hepatic encephalopathy by reducing gut-derived endotoxemia, preserving mucosal and intestinal epithelial integrity, and suppressing systemic inflammation [312]. Patients with MAFLD/MASH exhibit a decreased F/B ratio [313], along with a dysbiotic shift marked by increased abundances of Proteobacteria, *Enterobacteriaceae*, and *Escherichia* spp.—taxa associated with pro-inflammatory responses. At the same time, beneficial microbes such as *Faecalibacterium prausnitzii* and *Akkermansia muciniphila* were significantly depleted, further supporting the role of gut microbiota in the pathogenesis of liver diseases.

### 4.8. Hepatocellular Carcinoma

The gut–liver axis is linked via the hepatic portal venous system, exposing the liver to microbial products, including pathogens and metabolites, which in turn have been implicated in the pathogenesis of hepatocellular carcinoma (HCC), the most common primary liver cancer. Microbial metabolites, sensed by liver-resident immune cells, can trigger chronic inflammation and modulate cellular pathways, thereby contributing significantly to tumor development and progression [314]. A primary driver for HCC development is underlying chronic liver disease, with metabolic dysfunction-associated fatty liver disease (MAFLD), and particularly its progressive form, metabolic dysfunction-associated steatohepatitis (MASH), being increasingly recognized etiologies [315,316]. As detailed earlier in this review, gut dysbiosis is a pivotal contributor to MAFLD/MASH pathogenesis. This altered microbial state fosters a pro-carcinogenic liver environment. For instance, compositional differences in the gut microbiota are observed between HCC patients and healthy individuals; notably, an increased abundance of species like *Escherichia coli* has been reported in HCC patients [314]. Such dysbiosis, potentially leading to excessive microbial growth or an increase in pathobionts, is thought to promote liver cancer by exacerbating intestinal barrier dysfunction, increasing the translocation of pro-inflammatory microbial components like LPS, and altering local immune surveillance [317].

Dietary factors play a crucial role in shaping this interaction. A study investigating the impact of high dietary cholesterol, for example, demonstrated its capacity to induce HCC development and gut dysbiosis in mice, which exhibited a distinct gut microbiota profile [318], highlighting the influence of diet on HCC risk. Therapeutic modulation of gut microbiota is, therefore, an area of active investigation. Encouragingly, multi-strain probiotic supplementation in patients with MAFLD-HCC has demonstrated efficacy in mitigating the inflammatory response, evidenced by decreased TNF-α and IL-6 levels [319]. Such findings support the potential of targeted microbial interventions, with ongoing research exploring next-generation probiotics and engineered consortia for more precise effects.

Among the key microbial metabolites influencing HCC are bile acids. These endogenous signaling molecules are critical for maintaining liver and intestinal homeostasis, but disruptions in their complex metabolism and signaling are strongly linked to HCC development [320]. For example, deoxycholate (DCA), a major secondary bile acid produced by gut bacteria, can, when in excess, promote oncogenic processes by activating NF-κB, a key factor in apoptosis resistance, and inducing reactive oxygen species (ROS) that cause cellular damage [321]. Our previous work further underscored this by demonstrating that silent cholemia (elevated serum bile acids due to portosystemic shunting without overt clinical symptoms) may predispose individuals to liver injury and HCC, particularly when combined with a fermentable fiber-enriched diet like inulin [322]. These findings emphasize that both the quantity and composition of bile acids, influenced by diet and microbiota, are critical. With certain profiles being pro-tumorigenic while others might be protective, the dual role of bile acids makes their metabolism a key focus for novel therapeutic strategies [323]. Consequently, targeted dietary modifications and therapeutic interventions aimed at normalizing bile acid profiles and signaling pathways represent promising avenues for mitigating liver damage and preventing HCC progression.

### 4.9. Inflammatory Bowel Diseases

Inflammatory bowel diseases (IBD) are characterized by chronic inflammation of GI tissue, and most cases can be classified under ulcerative colitis (UC) or Crohn’s Disease (CD), depending on presentation and localization. The onset of IBD involves a host of factors, including genetic predispositions, environmental triggers, systemic health conditions, and gut microbiome activity. Breakdown of immune barrier function is a requisite first step in the onset of IBD, which is characterized by loss of Paneth and goblet cells and increased permeability of intestinal epithelial cells (IECs). Limited production of antimicrobial peptides and mucus secretions leaves IECs vulnerable to direct interaction with microbes. Compromised IECs lead to the recruitment of innate immune cells, primarily neutrophils and macrophages. While the immune response is largely similar, CD and UC differ in their activation of adaptive immunity, which is predominantly driven by Th1 and Th-17 responses in CD and characterized by Th2-like responses in UC [324].

Dysregulation of diet and microbiota are significant risk factors for the development of IBD. Elevated UPF consumption levels demonstrate robust associations with the risk of IBD development, with higher risk linked to specific subgroups, including sugar-sweetened beverages, salty snacks, refined sweetened foods, and processed meat products [136]. Additionally, patients reporting both HFD and antibiotic treatment were found to be at the highest risk of developing pre-IBD. HFD and antibiotic treatment work synergistically to contribute to dysbiosis by impairing mitochondrial bioenergetics and inducing Paneth and goblet cell dysfunction [177,325]. Reproducing this state of dysbiosis in the gut microbiome of offspring or via FMT results in significant worsening of colitis in mouse models [326]. Significant dysbiosis is both an indicator and a potential treatment route for IBD.

Functional dysbiosis in IBD patients is marked by a higher ratio of facultative to obligate anaerobes, depleted SCFA-producing species, higher *E. coli* and *Ruminococcus* levels, and lower *Faecalibacterium* and *Roseburia* species abundance [327]. Significant dysbiosis is an indicator of advanced IBD progression. Pediatric patients with acute severe colitis (ASC) demonstrate gut microbiomes with greater than 33% of total bacterial abundance attributable to a single species [328]. On the other hand, individual species have been highlighted in the literature for their robust protective activity against IBD. For example, UC patients with a lower abundance of *Faecalibacterium prausnitzii* were at significantly higher risk of relapse [329]. Isolation and characterization of *F. prausnitzii* supernatant components allowed investigators to directly attribute its anti-inflammatory and anti-colitic effects to butyrate production. Butyrate inhibits HDAC-1 and HDAC-3, blocking IL-6/STAT3/IL-17 signaling, ultimately promoting T-reg cells as opposed to Th-17 response [44,90,330]. The ability of butyrate to exert anti-inflammatory effects by inhibiting TLR-2 signal cascade and IL-12 and TNF-α production is impaired in IBD patients [331]. Lactic acid bacteria strains have also been shown to play a role in IBD amelioration. *Bifidobacterium adolescentis*, *Lactobacillus mucosae*, *Lactobacillus sakei*, and *Weissella cibaria* promote IL-10 expression, and oral gavage of these species significantly reduced experimentally induced colitis in mouse models [223,224]. Emerging evidence suggests that a reduced F/B ratio is associated with greater severity of IBD [332]. Notably, *Faecalibacterium prausnitzii*, is often depleted in IBD patients. Given these associations, the F/B ratio may serve as a useful biomarker for predicting disease progression and severity in IBD.

Comparative diet studies provide evidence that restricted carbohydrate, whole food, Mediterranean, and low-fat, high-fiber diets facilitate remission in IBD patients. Inflammatory markers often show significant improvement from baseline levels in dietary interventions, but the differences between specific diets are generally not substantial enough to identify a single gold standard [333,334]. A common constraint in IBD dietary studies is that simply adopting a more reliable and nutritious diet, regardless of its specific composition, tends to improve outcomes compared to typical baseline eating habits. Considering other metrics, the low-fat, high-fiber diet had positive effects on microbiome composition, increasing the abundance of established beneficial microbes such as *F. prausnitzii* [335]. Dietary restriction of fermentable oligosaccharides, disaccharides, monosaccharides, and polyols (FODMAPs) promotes normalization of microbiota composition and alleviates symptoms of GI discomfort in patients with quiescent IBD but falls short of improving IBD severity scores [336,337,338,339]. FODMAPs contribute to gastrointestinal symptoms by increasing osmotic potential and microbial fermentation activity in the gut. These results lead to greater water- and gas-induced distension and abdominal pain [340].

Dietary supplementation rather than restriction also has potential therapeutic benefits. Fructan supplementation in UC patients resulted in reduced inflammatory markers and increased butyrate production [70]. Oral butyrate supplementation has demonstrated upregulation of SCFA-producing bacterial species, as well as negative association with calprotectin levels, which act as a marker of neutrophil activity and IBD [82,341]. Honey-derived polyphenols downregulate the expression of genes facilitating IL-1b, IL-6, TNF-α, and IFN-γ inflammatory responses [106]. Polyphenol treatment significantly improved resistance to oxidative stress, an effect which was attributed to compositional rather than functional microbiome changes, with significant decreases in *Bacteroides*, *Corynebacterium,* and *Proteus* genera. The ketone body β-hydroxybutyrate (BHB) also exhibits an inverse relationship with IBD severity and alleviates experimentally induced IBD through activation of M2 macrophage-associated genes, but dietary modulation to promote BHB levels requires further study [342].

In addition to dietary factors, there are several gut microbiome-focused treatment strategies emerging for IBD. A small number of trials have investigated the ability of FMT to promote remission of UC. Study participants receiving FMT were more likely to achieve remission than control group participants, but further study is required to define the risk of adverse events [343]. In cases of *C. difficile* infection (CDI) secondary to IBD, FMT was effective in both eliminating CDI and clinical remission of IBD [344]. Finally, antibiotic cocktails have been proposed as a replacement or adjunct therapy alongside the current first-line treatment of intravenous corticosteroids [328,345,346]. While antibiotic-induced dysbiosis is a risk factor for IBD development, patients with severely imbalanced microbiomes, as seen in ASC, can benefit from broad-spectrum antibiotic suppression of bacterial load.

### 4.10. Colorectal Carcinoma

The gut microbiome is increasingly recognized as a critical player in colorectal carcinoma (CRC), influencing its development, progression, and even response to therapy. This complex community of microorganisms and their metabolites can contribute to carcinogenesis through mechanisms like inducing chronic inflammation and immune dysregulation [347]. Several microbial factors are implicated in CRC. Notably, pathogens such as *Helicobacter pylori* and hepatitis B virus have been associated with CRC development [348]. More broadly, an imbalance in the gut microbiota, or dysbiosis, can fuel chronic inflammation, a key driver of cancer. This occurs via multiple pathways, including the activation of NF-κB, Wnt signaling, and MAPK pathways, alongside the inhibition of apoptosis and increased oxidative stress [349]. Epigenetic alterations, like DNA methylation induced by microbial activities, can also perpetuate this pro-inflammatory state [350]. Furthermore, dysregulated immune responses, influenced by the microbiota, can contribute to tumor formation [351]. Microbial metabolites are also deeply involved, capable of promoting inflammation, aberrant cell proliferation, and DNA damage [352,353]. The composition of the gut microbiome in CRC patients often shows distinct alterations. There is typically a depletion of beneficial bacteria like *Clostridium butyricum* and *Streptococcus thermophilus*, which produce anti-carcinogenic substances such as butyrate [354]. Conversely, an increase in bacteria like *Fusobacterium nucleatum*, known for its role in other pathologies, is frequently observed [355]. For instance, lipopolysaccharide (LPS) receptor subunits on colonocytes can inhibit cell death and activate immune responses, leading to pro-inflammatory cytokine signaling that promotes tumorigenesis [356].

Diet significantly shapes the gut microbiome and, consequently, CRC risk. High-fat diets, for example, are linked to an increased production of tumorigenic secondary bile acids [357]. In contrast, plant-based diets can foster a microbiome profile that reduces inflammatory responses [23], while Western diets tend to promote pro-inflammatory microbial communities, elevating CRC risk [358]. Butyrate, a short-chain fatty acid produced from the fermentation of dietary fibers by microbes like *Faecalibacterium prausnitzii*, demonstrates anti-tumorigenic properties. It contributes to healthy energy balance, regulates colonic epithelial cell proliferation [359], and can inhibit histone deacetylase 3 (HDAC3), promoting the degradation of the proto-oncogene c-Myc [330].

Emerging research also highlights the potential for modulating the gut microbiome in CRC management. Postoperative probiotic administration in CRC patients undergoing chemotherapy has been shown to decrease gastrointestinal complications, particularly diarrhea. This intervention was associated with a shift in gut microbial composition, characterized by decreased Firmicutes and increased Bacteroidetes, Proteobacteria, and Verrucomicrobia [360]. Additionally, studies exploring the link between vitamin D levels and CRC risk have revealed that vitamin D supplementation can alter the gut microbiota, with outcomes potentially differing by sex. For instance, supplemented women were more likely to harbor *Fusobacterium nucleatum*, which is associated with shorter remission periods [361].

### 4.11. Celiac Disease

Celiac disease (CeD) is an autoimmune disease triggered when gluten absorbed in small intestinal tissues is bound by antigen-presenting cells, activating an inflammatory response and damaging the intestinal epithelium. The onset of CeD is known to be dependent on the possession of HLA-II DQ2 or DQ8 alleles and gluten peptide exposure, but other factors determining etiology remain under investigation [362]. Recent studies underscore the critical role of the gut microbiome in gluten metabolism and celiac disease pathogenesis. Several cultivable gut microbes, including certain *Lactobacillus* and *Clostridium* species, can metabolize gluten peptides, potentially reducing their immunogenicity and mitigating immune activation. However, not all microbial activity is protective. Some bacterial strains such as *Staphylococcus epidermidis*, *Enterococcus faecalis*, *Escherichia coli*, *Clostridium perfringens,* and *C. sordellii* may produce metabolites or enzymatic products that increase peptide immunogenicity or exacerbate intestinal inflammation. The presence of these non-beneficial bacteria may contribute to increased intestinal permeability, heightened immune responses, and more severe disease phenotypes. These findings emphasize the dualistic nature of microbiome–gluten interactions and the potential for targeted microbiome modulation in managing celiac disease [363].

Given its facilitation of nutrient processing and immune modulation, the gut microbiome has become a high-profile target for CeD treatment. Interestingly, a growing body of retroactive association studies shows a significant correlation between early exposure to systemic antibiotics and the risk of CeD [364,365]. Several hypotheses have been proposed to explain this association, including antibiotic-induced dysbiosis, inadequate microbial colonization, and disruption of mucosal barrier integrity [365]. Identifying consistent characteristics across CeD patient microbiomes may be useful in understanding microbe involvement in disease onset and altered gut function. Characterization studies have highlighted a reduced F/B ratio, as well as lower abundances of Actinobacteria and Euryarchaeota in CeD patients compared to healthy individuals [366]. In a longitudinal study following children with high genetic risk of CeD, however, there were no microbial signatures that accurately predicted which children would go on to develop the disease [367]. A higher relative abundance of *Bifidobacterium* has been suggested to have a causative relationship with the development of CeD, but underpinning mechanisms are still unknown [368].

Despite its associations with increased risk, probiotic formulations of *Bifidobacterium* species have demonstrated potential therapeutic benefits in the treatment of CeD patients. Supplementation of CeD patient diets with *Bifidobacterium breve* probiotics reinstated a normal F/B and led to relative increases in Actinobacteria abundance [366]. Daily administration of *Bifidobacterium breve* to children living with CeD and on a gluten-free diet yielded significant decreases in the levels of pro-inflammatory TNF-α over a three-month randomized controlled trial (RCT). The observed reduction in TNF-α levels was reversed for three months following the cessation of probiotic administration [369,370]. Verrucomicrobia and Parcubacteria abundance may play a role in this inflammatory modulation, as abundance in these phyla demonstrated a strong correlation with TNF-α levels in children with CeD [370]. *Bifidobacterium longum* probiotics have demonstrated mechanisms alleviating gluten-induced autoimmune response, potentially through serine protease inhibition, as marked by lower fecal sIgA content levels [371,372]. Meta-analysis of RCTs investigating probiotic treatment efficacy over placebo in cases of CeD paints a complicated picture. Evidence of symptomatic improvement remains inconclusive, but probiotics demonstrate the ability to increase potentially therapeutic *Bifidobacterium* and *Lactobacillus* species, leaving the door open to their role in helping normalize dysbiotic microbiomes [373,374].

The primary treatment of CeD is lifetime adherence to a strict gluten-free diet (GFD). A gluten-free diet significantly reduces macrophage counts, promotes epithelial integrity, and improves subjective wellbeing but does not facilitate significant improvement in small bowel water content or whole gut transit time after a year [375,376]. Additionally, GFD is associated with sharp reductions in *Bifidobacterium* species and loss of starch and arabinoxylan degrading enzyme activity [376]. Further dietary modifications to GFD have the potential to potentially improve symptom severity and prevent dysbiosis. CeD patients adopting a low-FODMAP diet, on top of gluten restriction, showed statistically significant improvements in pain, bloating, diarrhea, and satiety within 4 weeks [377]. Recent investigations of commensal *Lactobacillus* species have outlined mechanisms for degrading and decreasing the immunogenicity of gluten molecules, reducing residual gluten levels in healthy volunteers [378,379]. Probiotic formulations taking advantage of these mechanisms demonstrate therapeutic potential in reducing the consequences of gluten cross-contamination for CeD patients. While the role of gut microbiota in CeD etiology remains to be accurately defined, modulating gut microbiota activity through diet and supplementation has the potential to improve the efficacy of CeD treatment. 

### 4.12. Rheumatoid Arthritis

Rheumatoid arthritis (RA) is a chronic autoimmune disease predominantly targeting the joints, with microbial infections increasingly recognized as potential triggers [380]. Emerging evidence strongly implicates the composition and function of the gut microbiota and its dysbiosis in the complex immune dysregulation characteristic of RA, although the precise etiological and pathogenetic pathways continue to be actively investigated [381]. Several mechanisms have been proposed to explain how the gut microbiota may contribute to RA pathogenesis. These include the production of pro-inflammatory metabolites by certain gut bacteria, the impairment of the intestinal mucosal barrier, and molecular mimicry, where microbial antigens resemble host autoantigens, potentially initiating or perpetuating an autoimmune response [380]. Patients with RA often exhibit an imbalance in cytokine profiles, with an upregulation of pro-inflammatory cytokines and a downregulation of anti-inflammatory ones, a milieu potentially influenced by microbial factors [382]. Compromised intestinal mucosal permeability can lead to the translocation of microbial products and even whole bacteria into circulation. This breach is thought to facilitate the migration of autoreactive immune cells from the gut to the joints, thereby contributing to synovial inflammation [383].

While the exact nature of microbial variance in RA patients remains a subject of ongoing research and debate [384,385,386], specific microbial signatures have been associated with the disease. Notably, increased relative abundances of species like *Prevotella copri* and certain *Lactobacillus* strains, alongside decreased levels of Bacteroidetes, *Bifidobacteria*, and *Eubacterium rectale*, have been reported, particularly in the early stages of RA [387]. More recent research comparing RA patients undergoing sustained drug treatment with untreated patients and healthy controls has highlighted differences in microbial metabolic pathways. For instance, pathways such as vitamin K2 biosynthesis were found to be more abundant in bacteria enriched in untreated RA patients, suggesting functional consequences of the observed dysbiosis [388]. Given that RA is characterized by heightened inflammation and oxidative stress, interventions targeting gut microbiota have become an area of significant interest. RA patients receiving probiotics have demonstrated improvements in their oxidative/nitrosative stress profiles and reductions in inflammatory biomarkers compared to those receiving a placebo. These findings suggest that modulating gut microbiome diversity through probiotics can foster a beneficial immune response and decrease systemic inflammation in RA [384].

Dietary modulation also presents a compelling therapeutic strategy. Investigations into various dietary patterns have found that the Mediterranean diet, rich in anti-inflammatory components, may offer greater efficacy in ameliorating the perception of pain in RA patients compared to other approaches like general plant-based diets [389,390,391]. While larger, blinded patient cohorts are necessary to solidify the connection between specific diets, gut microbiota changes, and RA-associated pain relief, this growing body of research underscores the significant potential of dietary recommendations to improve the quality of life for individuals living with RA.

### 4.13. Neurological Disorders

The role of the gut–microbiome–brain axis is emerging as an important consideration in the pathogenesis and treatment of several neurological conditions. The bidirectional nature of gut–brain interaction has been emphasized in recent studies outlining spinal cord injury-induced gut microbe dysbiosis. Spinal cord injury was found to interrupt signaling from the brain to sympathetic preganglionic neurons, resulting in a loss of coordinated control over GI tissues, with subsequent disruptions of motility, mucous secretion, epithelial barrier permeability, and immune surveillance [392]. The resulting compositional changes and immune activation resulted in delayed recovery from neurological and locomotor impairment [393]. Mice were administered LAB probiotics following spinal cord injury; however, they demonstrated improved locomotor recovery time. Communication along the gut–brain axis is mediated by a complex network of microbiota-mediated metabolite and cytokine interactions with implications for the etiology and progression of neurological diseases.

Gut microbe secretion of SCFAs has been demonstrated to play a key role in the modulation of neuroinflammatory processes. Another notable biological compound prominently active in neurological disorders is tryptophan, whose metabolites are linked to the secretion and regulation of serotonin and melatonin, which are closely linked to several neurological disorders [394]. Key markers associated with gut dysbiosis for many neurological disorders have been identified in a systematic review, noting consistently increased circulating levels of tight-junction protein zonulin, the endotoxin LPS and gut-related systematic inflammatory markers [395].

Major depressive disorder (MDD) has similarly been linked to gut microbiota dysbiosis. The gut microbiome of MDD patients is characterized by an increased abundance of *Enterococcus*. The immune systems of individuals with MDD show a distinct profile with increased pro-inflammatory and decreased anti-inflammatory immune cell subsets, highlighting the immune system’s involvement in the disorder [396]. The crosslink between the gut, the disease, and the immune system points to possible advancement in a more comprehensive understanding of the disease. There is now research being conducted to investigate the possible adjunct therapy use of probiotics to treat or prevent MDD. Research correlated high levels of *Bifidobacterium* with stress-resistant traits in mice and has seen positive results of the supplementation of the bacteria, having a possible preventative nature and resilience to stress. Administering *Lactobacillus* or *Bifidobacterium* spp. has been shown to lower cortisol levels in acute or chronic stress in mice [397]. Further implicating the brain–gut axis in MDD, a recent study explored the anti-depressant effects of ginsenoside Rh4 and its mechanisms of action, showing alongside its effectiveness in treating depression-like behavior in a mouse model, it has concurrently shown to inhibit hippocampal neuronal apoptosis and synaptic structural damage from proinflammatory cytokines alongside increasing the SCFA content [398]. Although there are numerous studies linking alleviated symptoms of MDD to the Mediterranean diet, the exact mechanism that shows causality is still in question and potential for further research [399,400,401].

Social anxiety disorder (SAD) is a debilitating psychiatric disorder associated with intense fear or anxiety in social situations. Fecal microbiota transplantation (FMT) from mice with a model of SAD to healthy control mice induced behaviors resembling heightened social fear, suggesting a causal role for the gut microbiota in SAD-like behavior [402]. This link opens opportunities to look for possible therapeutics to alleviate the heightened fear symptoms of SAD. A recent randomized controlled trial investigating the effects of *Lactobacillus plantarum* JYLP-326 on test anxious college students resulted in alleviation of anxiety, depression, and insomnia after taking this probiotic and further highlighting the connection between gut microbiota and mental health [403].

Recent clinical studies have demonstrated that FMT may offer therapeutic benefits for children with autism spectrum disorder (ASD). Multiple trials have reported improvements in gastrointestinal symptoms as well as reductions in core behavioral symptoms of ASD following FMT treatment, suggesting a direct role of the gut microbiome in modulating neurological function and symptom severity over time [404,405]. These findings support the hypothesis of a gut–brain axis involvement in ASD pathogenesis and highlight the potential of microbiome-targeted therapies, such as FMT, as promising interventions for ameliorating disease symptoms.

Alzheimer’s disease (AD), a neurodegenerative condition characterized by the buildup of extracellular amyloid plaques and sustained neuroinflammation, has a growing body of evidence underscoring the critical involvement of the gut–microbiota–brain axis and immune system alterations in AD pathogenesis. Dietary patterns high in ultra-processed foods (UPFs) show detrimental associations; for instance, over 10 years, a 10% increase in UPF consumption was associated with an increased risk of dementia and AD, while replacing 10% of dietary UPFs with minimally processed foods lowered dementia risk by nearly 20% [406]. Changes in the gut microbial composition are observed in AD. Notably, AD correlates with significantly reduced short-chain fatty acids. The strongest known genetic risk factor for late-onset AD, the Apolipoprotein E4 allele, also appears to intersect with the gut microbiome. Research indicates that the genotype is associated with distinct gut microbiome profiles compared to non-carriers. These alleles-linked alterations in microbial composition may foster a pro-inflammatory gut environment, further contributing to the systemic inflammation and immune dysregulation implicated in AD. This emerging understanding suggests that the detrimental effects of the allele may not be solely confined to its roles in central nervous system metabolism and lipid transport but may also be mediated through its influence on the gut microbiota. Such findings are pivotal, opening new avenues for developing therapeutic strategies that specifically target the gut microbiome to potentially mitigate AD risk or slow its progression, particularly in genetically susceptible individuals [407]. New and unique therapeutic approaches targeting the microbiota–gut–brain axis are emerging, reflecting this paradigm shift. For instance, interventions such as acupuncture have garnered attention for their potential neuroprotective effects. Studies have reported that acupuncture treatment can improve cognitive function and favorable modulations in brain activity in AD models and patients. Crucially, these benefits have been linked to concurrent positive changes in the composition and functional capacity of the gut microbiota. These observations further solidify the hypothesis that the microbiota–gut–brain axis plays a role in AD pathophysiology and highlight its potential as a viable target for innovative treatment strategies aimed at restoring microbial symbiosis and dampening detrimental immune responses [408].

Metagenomic investigation of Parkinson’s disease (PD) patients revealed a unique gut microbiota profile, leading investigators to successfully supplement changes in the gut to alleviate PD symptoms [409]. The relationship between the altered gut and the pathogenesis of PD may be linked to the decreased levels of anti-inflammatory SCFAs and subsequent upregulation of inflammatory response. FMT as a treatment for PD has so far shown limited clinically meaningful improvements [410]. However, these findings emphasize the need for further investigation into the specific mechanisms underlying the gut–microbiota–brain axis in PD. One common non-motor symptom in PD impairing the quality of life of the patient is constipation. In hopes of improving constipation symptoms in PD patients, a recent study supplemented patients with probiotics and has seen this treatment effectively improve the symptoms and positively affect the gut microbiota [411]. A healthy and balanced diet plays a critical role in shaping microbial diversity in the gut, which leads to higher production of SCFA and other beneficial biological compounds that positively affect the brain processes [412]. Although the understanding of the gut–microbiota–brain axis is still in its early stages, the foundational research linking neurological diseases to gut health underscores the intricate interconnectedness of bodily functions and provides a promising starting point for future discoveries.

## 5. Conclusions

The impact of diet on the gut microbiota–immune axis underscores the importance of research on its therapeutic potential in many diseases. Interventional diets have consistently been shown to reduce inflammation, protect the gut barrier, and aid immune regulation. The Mediterranean diet, increased fiber and polyphenols, balancing diversity, and limiting high fat and sugar have all demonstrated positive modulating effects on the microbiome. Microbial metabolites, e.g., SCFA, secondary bile acids, and TMAO, alongside microbial structural components, e.g., LPS, are critical in the crosstalk between the gut and systemic processes, serving as key players in disease etiology and pathogenesis (Figure 1).

Alterations in microbial diversity and function are consistently linked to a wide spectrum of diseases, including cardiometabolic disorders (e.g., cardiovascular disease, obesity, type-2 diabetes), autoimmune conditions (e.g., type-1 diabetes, rheumatoid arthritis, celiac disease), respiratory disease, chronic kidney and liver disease, inflammatory bowel disease, and neurological disorders (e.g., Alzheimer’s, Parkinson’s disease, depressive disorders). The specific mechanisms implicating gut dysbiosis in these various diseases are multifaceted and intertwined, often involving chronic low-grade inflammation, impaired metabolic function, gut barrier dysfunction, and immune dysregulation. Furthermore, the extension of the microbiome’s influence in regulating host circadian rhythms adds an additional layer of complexity in affecting the systemic processes and diseases.

FMT has emerged as an important therapeutic approach for addressing gut dysbiosis. By restoring a healthy and diverse microbial community, FMT can influence immune regulation, helping to modulate inflammatory responses and improve disease outcomes. In various disease pathogenesis, including IBD, metabolic disorders, and even neurological conditions, FMT has shown promise in reshaping the microbiome and reducing inflammation. Moreover, the functional capacity of the gut microbiome to metabolize a wide range of therapeutic drugs is increasingly recognized as a key factor influencing clinical outcomes. Notably, Zimmermann et al. (2019) demonstrated that specific gut bacterial genes could significantly alter drug metabolism, underscoring the need to consider microbial composition and function in personalized medicine [413].

While the loss of beneficial microbes is often associated with disease states, their absence may not universally contribute to pathogenesis. In some conditions, host genetics or environmental exposures may dominate disease etiology. For example, certain acute infections or monogenic disorders may proceed independently of microbial contributions [414]. Moreover, microbial resilience or functional redundancy within the microbiome may compensate for the loss of specific beneficial strains. These exceptions highlight the importance of contextualizing microbiome-disease associations within a broader host and environmental frameworks [415,416].

Despite significant advances, challenges remain, as the high inter-individual variability in microbiome composition and differential response to interventions highlight the need for personalized approaches. A more complete and nuanced understanding of the complex molecular mechanisms underlying host–microbe interactions in different disease contexts is crucial for developing targeted and effective therapies. Future research should focus on identifying reliable biomarkers of dysbiosis, elucidating causal relationships between specific microbial taxa or functions and disease progression, and conducting clinical trials to validate the efficacy and safety of microbiome-modulating strategies. Incorporating diet to cultivate a health-promoting gut microbiome and a balanced immune system is a crucial component of the push for a more complete understanding of this dynamic network, holding immense promise for revolutionizing the prevention and management of various diseases.

## Abbreviations

AD	Alzheimer’s disease
ALT	Alanine aminotransferase
AMPs	Antimicrobial proteins
ANGPTL4	Angiopoietin-like protein 4
APC	Antigen-presenting cell
ARG	Antibiotic resistance genes
ASD	Autism spectrum disorder
AST	Aspartate aminotransferase
ASC	Acute severe colitis
BCFAs	Branched-chain fatty acids
BHB	Beta (β) hydroxybutyrate
CD	Crohn’s Disease
CKD	Chronic kidney disease
CLA	Conjugated linoleic acid
CVD	Cardiovascular disease
DCA	Deoxycholic acid
DCs	Dendritic cells
D-PLA	D-phenyllactic acid
FMT	Fecal microbiota transplantation
FMT	Fecal microbiota transplantation
GALT	Gut-associated lymphoid tissues
GF	Germ-free
GFD	Gluten free diet
GI	Gastrointestinal tract
GLP-1	Glucagon-like peptide-1
GPCRs	G-protein-coupled receptors
HCA3	Hydroxycarboxylic acid
HDAC	Histone deacetylase
HDACs	Histone deacetylases
HFD	High-fat diet
IBD	Inflammatory bowel diseases
IECs	Intestinal epithelial cells
ILCs	Innate lymphoid cells
ILFs	Isolated lymphoid follicles
LAB	Lactic acid bacteria
LP	Lamina propria
LPS	Lipopolysaccharide
MACs	Microbiota accessible carbohydrates
MAMPs	Microbe-associated molecular patterns
MDD	Major depressive disorder
Mφ	Macrophages
MTB	*Mycobacterium tuberculosis*
NADPH	Nicotinamide-adenine dinucleotide phosphate
MAFLD	Metabolic dysfunction-associated fatty liver disease
MASH	Metabolic dysfunction-associated steatohepatitis
NF-kB	Nuclear factor kappa-light-chain-enhancer of activated B cells
NK	Natural killer
PD	Parkinson’s disease
PPs	Peyer’s patches
PRRs	Pattern-recognition receptors
PYY	Plasma peptide YY
RAAS	Renin–angiotensin–aldosterone system
ROS	Reactive oxygen species
SAD	Social anxiety disorder
SCFAs	Short-chain fatty acids
SCN	Suprachiasmatic nucleus
TB	Tuberculosis
TCR	T cell receptor
TLR	Toll-like receptor
TMA	Trimethylamine
TMAO	Trimethylamine-N-oxide
UC	Ulcerative colitis
UPF	Ultra-processed food
